# Subdivision of the MDR superfamily of medium-chain dehydrogenases/reductases through iterative hidden Markov model refinement

**DOI:** 10.1186/1471-2105-11-534

**Published:** 2010-10-27

**Authors:** Joel Hedlund, Hans Jörnvall, Bengt Persson

**Affiliations:** 1 IFM Bioinformatics, Linköping University, S-581 83 Linköping, Sweden; 2 Dept of Medical Biochemistry and Biophysics, Karolinska Institutet, S-171 77 Stockholm, Sweden; 3 Dept of Cell and Molecular Biology, Karolinska Institutet, S-171 77 Stockholm, Sweden; 4 National Supercomputer Centre (NSC) and Swedish E-science Research Centre (SERC), Linköping University, S-581 83 Linköping, Sweden

## Abstract

**Background:**

The Medium-chain Dehydrogenases/Reductases (MDR) form a protein superfamily whose size and complexity defeats traditional means of subclassification; it currently has over 15000 members in the databases, the pairwise sequence identity is typically around 25%, there are members from all kingdoms of life, the chain-lengths vary as does the oligomericity, and the members are partaking in a multitude of biological processes. There are profile hidden Markov models (HMMs) available for detecting MDR superfamily members, but none for determining which MDR family each protein belongs to. The current torrential influx of new sequence data enables elucidation of more and more protein families, and at an increasingly fine granularity. However, gathering good quality training data usually requires manual attention by experts and has therefore been the rate limiting step for expanding the number of available models.

**Results:**

We have developed an automated algorithm for HMM refinement that produces stable and reliable models for protein families. This algorithm uses relationships found in data to generate confident seed sets. Using this algorithm we have produced HMMs for 86 distinct MDR families and 34 of their subfamilies which can be used in automated annotation of new sequences. We find that MDR forms with 2 Zn^2+ ^ions in general are dehydrogenases, while MDR forms with no Zn^2+ ^in general are reductases. Furthermore, in Bacteria MDRs without Zn^2+ ^are more frequent than those with Zn^2+^, while the opposite is true for eukaryotic MDRs, indicating that Zn^2+ ^has been recruited into the MDR superfamily after the initial life kingdom separations. We have also developed a web site http://mdr-enzymes.org that provides textual and numeric search against various characterised MDR family properties, as well as sequence scan functions for reliable classification of novel MDR sequences.

**Conclusions:**

Our method of refinement can be readily applied to create stable and reliable HMMs for both MDR and other protein families, and to confidently subdivide large and complex protein superfamilies. HMMs created using this algorithm correspond to evolutionary entities, making resolution of overlapping models straightforward. The implementation and support scripts for running the algorithm on computer clusters are available as open source software, and the database files underlying the web site are freely downloadable. The web site also makes our findings directly useful also for non-bioinformaticians.

## Background

The superfamily of MDR proteins is formed by quinone reductases, leukotriene B4 dehydrogenases, polyol dehydrogenases, Zn^2+ ^dependent ADHs (alcohol dehydrogenases), and many more families. A recent estimate places the total number of MDR families close to 500, with less than 30% sequence identity between the families [1]. Numbers of known superfamily members have grown considerably in recent years, and it currently has over 15000 members in the UniProtKB database [2], which is more than an order of magnitude higher than the number six years ago [3]. Disregarding species variants, there is considerable multiplicity, with at least 25 MDR forms in the human, excluding close homologues. Roughly half of the MDR proteins can be grouped into large clusters with 100's of members, while about 1000 forms belong to small clusters with 10 or fewer members each. Thus, the MDR family is now showing a spread resembling the complexity of the SDR (short-chain dehydrogenase/reductase) family [4]. This is a new characteristic of the MDR family, not detectable until now when data from many large-scale genome projects exist.

The first characterised member was the class I type of mammalian alcohol dehydrogenase, for which the primary structure was reported in 1970 [5]. Subsequently detected MDR members included sorbitol dehydrogenase [6], a crystallin, metabolic enzymes and a synaptic protein [7]. The latter report also coined the term MDR [7]. This distinction from SDR, with its typically smaller subunits (~250-residue subunits) and no metal dependence, was known [8], but initially named as a long-chain form. The split into separate protein types was detected already in the 1970's when the first data on *Drosophila *ADH showed sequence patterns separate from those of the Zn^2+ ^dependent ADHs [9].

The MDR proteins typically consist of two domains, where the C-terminal domain is coenzyme-binding with the ubiquitous Rossmann fold [10] of an often six-stranded parallel *β*-sheet sandwiched between *α*-helices on each side. The N-terminal domain is substrate binding with a core of antiparallel *β*-strands and surface-positioned *α*-helices, showing distant homology with the GroES structure [11]. The domains are separated by a cleft containing a deep pocket which accommodates the active site. The MDR proteins generally form homodimers, but some members are active as monomers like the MTD family [12,13], or tetramers like the PDH family. The metal dependence also varies between families. While many MDR families bind one catalytic and one structural Zn^2+ ^per subunit [1], many sorbitol dehydrogenases in the PDH family bind only the catalytic Zn^2+ ^[6,14,15] (cf. MDR005 below), and the prostaglandin reductases in the PTGR family bind no Zn^2+ ^[16].

In a recent review [1] we showed that the size and complexity of the MDR superfamily challenges traditional means of subclassification such as linear sequence pattern matching, but we were able to present models for automated classification of a number of the MDR families.

Hidden Markov models (HMMs) are statistical descriptions of the characteristic sequence variations within a protein family, and have been put to good use in protein classification since over a decade [17]. An alignment between a sequence and an HMM gives a statistical measure of the quality of the match, which can be used to reliably determine whether or not the sequence is a member of the modelled family. Collections of HMMs such as the PfamA and Interpro databases [18-20] therefore greatly facilitate the annotation of new sequences. Large families can also be subclassified using HMMs, which was recently demonstrated for the SDR superfamily of short-chain dehydrogenases/reductases [21].

HMMs are products of machine learning and the quality of the training data is therefore crucial for the reliability of resulting models. High quality training data should well describe the diversity within the family, without overgeneralisation that would result in overprediction. Assembling good quality training data usually requires hands-on work by experts in the field and has therefore been the rate limiting step for expanding the number of available protein family models. These principles also apply when HMMs are used for subclassification.

So far, there are HMMs available for detecting membership of the MDR superfamily, but no HMMs are available for the individual families. In this work, we present a method for automated HMM refinement that produces stable and reliable HMMs. We have used this method to produce HMMs for 86 distinct MDR families and 34 of their subfamilies. We have also characterised these families based on conservation, NAD(P) and Zn^2+ ^cofactor preference, and species distribution.

## Results and Discussion

### MDR families

Hidden Markov models (HMMs) were developed for protein families within the superfamily of MDR. This is necessary since the MDR superfamily now is found to be very large and divergent with over 15000 members and many different enzyme activities. In Pfam [20], there are only two MDR-HMMs available - one for the N-terminal domain [PfamA:PF08240] and one for the C-terminal domain [PfamA:PF00107]. The aim was that each HMM now developed should be specific for each MDR family. Therefore a method was developed to automatically derive stable HMMs using an iterative procedure.

Here, initial HMMs were built from multiple sequence alignments (MSAs) of disjoint MDR sequence clusters sharing more than 40% residue identities in pairwise comparisons. These HMMs were used to search the UniProtKB database finding additional members, which were then incorporated into their respective training sets to produce refined HMMs. After multiple iterations, typically below 8, the refined HMMs no longer detected any new members in the database. The stability of the model was assessed using jackknifing (cf. Methods), and any unstable HMMs were again subjected to iterative refinement once the spurious sequence(s) had been removed. After 4 reiterations, all except 2 HMMs were deemed stable and reliable (cf. Methods).

Using this iterative procedure, a total of 86 HMMs were developed, which are listed in Table 1. We assigned identifiers to these MDR families from MDR001 through MDR086 based on enumeration by decreasing family size as found in the present investigation, starting with families having at least one human representative and then families having at least one eukaryotic representative, and ending with the purely prokaryotic families. These HMMs encompass a total of 11579 members, thus representing just over 76% of all MDR superfamily members. The remaining members form families with too few members to establish HMMs with sufficiently strong predictive power. The 86 HMMs now presented will be helpful in assignment of functions for MDR members for which the enzymatic function is not known, and they will also help to increase the level of granularity attainable in reliable automatic functional assignment of new sequences. This is of course crucial for understanding relationships in new collected sequence data.

**Table 1:** Properties of MDR families

ID	Name	Size	Swiss-Prot	PCID Avg (StdDev)	Eukaryotic	Bacterial	Archaeal
MDR001	ADH	2217	116	51.24 (19.02)	1315 (59.3%)	895 (40.4%)	6 (0.3%)
MDR002	PTGR	774	17	42.46 (9.64)	253 (32.7%)	518 (66.9%)	3 (0.4%)
MDR003	FAS	706	11	39.07 (9.88)	288 (40.8%)	418 (59.2%)	0 (0.0%)
MDR004	QORX	486	1	47.47 (10.07)	69 (14.2%)	417 (85.8%)	0 (0.0%)
MDR005	PDH	328	18	45.59 (11.81)	181 (55.2%)	146 (44.5%)	1 (0.3%)
MDR006	ZADH2	56	4	51.49 (15.19)	51 (91.1%)	5 (8.9%)	0 (0.0%)
MDR007	MECR	51	9	54.60 (13.15)	51 (100.0%)	0 (0.0%)	0 (0.0%)
MDR008	VAT1	50	6	60.49 (19.60)	48 (96.0%)	2 (4.0%)	0 (0.0%)
MDR009	vertQOR	22	8	70.12 (10.34)	21 (95.5%)	1 (4.5%)	0 (0.0%)

MDR010	CAD	661	32	49.08 (10.02)	329 (49.8%)	330 (49.9%)	2 (0.3%)
MDR011	bpQOR	575	5	47.93 (11.86)	66 (11.5%)	509 (88.5%)	0 (0.0%)
MDR012	YHDH	481	2	53.92 (11.43)	1 (0.2%)	477 (99.2%)	0 (0.0%)
MDR013	FDH	375	4	47.16 (12.56)	29 (7.7%)	346 (92.3%)	0 (0.0%)
MDR014	TDH	351	126	69.40 (16.11)	1 (0.3%)	343 (97.7%)	7 (2.0%)
MDR015	QORL2	319	4	45.25 (8.76)	38 (11.9%)	281 (88.1%)	0 (0.0%)
MDR016	bADH	313	17	60.72 (15.62)	1 (0.3%)	312 (99.7%)	0 (0.0%)
MDR017		279	8	51.97 (8.93)	2 (0.7%)	277 (99.3%)	0 (0.0%)
MDR018		194	1	47.22 (10.60)	48 (24.7%)	146 (75.3%)	0 (0.0%)
MDR019		136	0	60.51 (12.37)	18 (13.2%)	118 (86.8%)	0 (0.0%)
MDR020	yADH	128	22	63.56 (12.11)	128 (100.0%)	0 (0.0%)	0 (0.0%)
MDR021	YJGB	122	1	69.98 (20.96)	2 (1.6%)	120 (98.4%)	0 (0.0%)
MDR022	giFDH	122	2	63.32 (18.53)	5 (4.1%)	111 (91.0%)	6 (4.9%)
MDR023		72	5	52.93 (17.12)	10 (13.9%)	56 (77.8%)	6 (8.3%)
MDR024		40	0	52.66 (11.72)	7 (17.5%)	32 (80.0%)	1 (2.5%)
MDR025	yPDH	34	0	65.44 (13.11)	34 (100.0%)	0 (0.0%)	0 (0.0%)
MDR026	yDH	34	0	60.48 (13.13)	34 (100.0%)	0 (0.0%)	0 (0.0%)
MDR027	QORH	34	2	60.28 (10.85)	34 (100.0%)	0 (0.0%)	0 (0.0%)
MDR028		29	0	55.36 (9.11)	1 (3.4%)	28 (96.6%)	0 (0.0%)
MDR029	yADH2	29	1	59.02 (15.24)	29 (100.0%)	0 (0.0%)	0 (0.0%)
MDR030	dFAS	28	0	57.51 (16.34)	28 (100.0%)	0 (0.0%)	0 (0.0%)
MDR031	yDH2	28	0	53.43 (12.87)	28 (100.0%)	0 (0.0%)	0 (0.0%)
MDR032	bADH2	26	0	69.59 (17.08)	1 (3.8%)	25 (96.2%)	0 (0.0%)
MDR033	yBDH	23	2	54.51 (12.62)	23 (100.0%)	0 (0.0%)	0 (0.0%)
MDR034		23	0	54.57 (14.09)	13 (56.5%)	10 (43.5%)	0 (0.0%)
MDR035		22	0	47.17 (10.55)	22 (100.0%)	0 (0.0%)	0 (0.0%)
MDR036	bADH3	21	4	50.26 (15.82)	1 (4.8%)	12 (57.1%)	8 (38.1%)
MDR037		20	0	66.14 (13.09)	20 (100.0%)	0 (0.0%)	0 (0.0%)
MDR038		20	0	74.56 (12.47)	20 (100.0%)	0 (0.0%)	0 (0.0%)

MDR039	bADH4	150	0	58.99 (10.83)	0 (0.0%)	150 (100.0%)	0 (0.0%)
MDR040	SORE	117	2	61.62 (21.91)	0 (0.0%)	117 (100.0%)	0 (0.0%)
MDR041	BurkDH	114	0	63.89 (15.74)	0 (0.0%)	114 (100.0%)	0 (0.0%)
MDR042	YJJN	114	1	57.50 (18.31)	0 (0.0%)	114 (100.0%)	0 (0.0%)
MDR043	IDND	96	1	55.99 (17.90)	0 (0.0%)	96 (100.0%)	0 (0.0%)
MDR044		96	0	54.24 (13.62)	0 (0.0%)	92 (95.8%)	4 (4.2%)
MDR045		89	0	56.57 (14.09)	0 (0.0%)	89 (100.0%)	0 (0.0%)
MDR046	RSPB	78	1	72.88 (16.80)	0 (0.0%)	78 (100.0%)	0 (0.0%)
MDR047		72	0	58.49 (18.18)	0 (0.0%)	72 (100.0%)	0 (0.0%)
MDR048	GATD	71	2	83.33 (14.94)	0 (0.0%)	71 (100.0%)	0 (0.0%)
MDR049		69	0	54.89 (14.55)	0 (0.0%)	69 (100.0%)	0 (0.0%)
MDR050	CCR	67	0	72.88 (15.83)	0 (0.0%)	67 (100.0%)	0 (0.0%)
MDR051	TARJ	63	1	60.87 (21.70)	0 (0.0%)	62 (98.4%)	1 (1.6%)
MDR052	YCJQ	48	2	91.08 (10.57)	0 (0.0%)	48 (100.0%)	0 (0.0%)
MDR053		48	0	50.84 (14.22)	0 (0.0%)	48 (100.0%)	0 (0.0%)
MDR054	YDJL	46	1	93.70 (12.44)	0 (0.0%)	46 (100.0%)	0 (0.0%)
MDR055		44	0	63.84 (10.29)	0 (0.0%)	44 (100.0%)	0 (0.0%)
MDR056		44	0	63.41 (20.64)	0 (0.0%)	44 (100.0%)	0 (0.0%)
MDR057	BCHC	43	2	61.89 (8.07)	0 (0.0%)	43 (100.0%)	0 (0.0%)
MDR058		43	0	76.53 (11.52)	0 (0.0%)	43 (100.0%)	0 (0.0%)
MDR059		42	0	56.97 (9.37)	0 (0.0%)	42 (100.0%)	0 (0.0%)
MDR060	YPHC	42	1	84.08 (22.01)	0 (0.0%)	42 (100.0%)	0 (0.0%)
MDR061	bBDH	40	0	63.33 (22.56)	0 (0.0%)	40 (100.0%)	0 (0.0%)
MDR062	CCR2	40	0	73.01 (14.76)	0 (0.0%)	40 (100.0%)	0 (0.0%)
MDR063		38	0	58.21 (20.04)	0 (0.0%)	38 (100.0%)	0 (0.0%)
MDR064		33	0	81.63 (18.31)	0 (0.0%)	33 (100.0%)	0 (0.0%)
MDR065		32	0	53.79 (11.72)	0 (0.0%)	31 (96.9%)	1 (3.1%)
MDR066		32	1	82.45 (18.25)	0 (0.0%)	32 (100.0%)	0 (0.0%)
MDR067	bQOR	32	0	73.92 (21.12)	0 (0.0%)	32 (100.0%)	0 (0.0%)
MDR068		32	0	55.05 (10.93)	0 (0.0%)	32 (100.0%)	0 (0.0%)
MDR069		32	0	54.76 (13.41)	0 (0.0%)	30 (93.8%)	2 (6.2%)
MDR070		31	0	50.14 (19.08)	0 (0.0%)	31 (100.0%)	0 (0.0%)
MDR071		31	0	52.17 (11.37)	0 (0.0%)	31 (100.0%)	0 (0.0%)
MDR072	bDHSO	31	1	65.28 (20.13)	0 (0.0%)	31 (100.0%)	0 (0.0%)
MDR073	bQOR2	31	0	81.84 (19.40)	0 (0.0%)	31 (100.0%)	0 (0.0%)
MDR074		30	0	79.07 (19.18)	0 (0.0%)	30 (100.0%)	0 (0.0%)
MDR075		29	0	82.92 (18.37)	0 (0.0%)	29 (100.0%)	0 (0.0%)
MDR076		29	0	57.41 (13.46)	0 (0.0%)	29 (100.0%)	0 (0.0%)
MDR077		28	0	58.92 (10.41)	0 (0.0%)	24 (85.7%)	4 (14.3%)
MDR078	RhobDH	25	0	55.27 (11.23)	0 (0.0%)	25 (100.0%)	0 (0.0%)
MDR079		25	0	63.69 (12.82)	0 (0.0%)	25 (100.0%)	0 (0.0%)
MDR080	bPDH	24	0	75.24 (15.12)	0 (0.0%)	24 (100.0%)	0 (0.0%)
MDR081		23	0	53.96 (19.57)	0 (0.0%)	23 (100.0%)	0 (0.0%)
MDR082		23	0	63.98 (10.48)	0 (0.0%)	23 (100.0%)	0 (0.0%)
MDR083		21	0	57.76 (20.40)	0 (0.0%)	9 (42.9%)	12 (57.1%)
MDR084		21	0	44.68 (12.83)	0 (0.0%)	21 (100.0%)	0 (0.0%)
MDR085		21	0	68.74 (15.71)	0 (0.0%)	21 (100.0%)	0 (0.0%)
MDR086	MycDH	20	0	65.99 (28.08)	0 (0.0%)	20 (100.0%)	0 (0.0%)

This table shows properties of the MDR families for which we have derived HMMs, including its assigned name (if any), size, number of members in the Swiss-Prot database, average percent pairwise sequence identities in the family (and sample standard deviation) and distribution of members over the kingdoms of life (and proportions). See also Additional file 2: mdr-properties for further data. Empty name fields indicate families where the function has not yet been established for any of the members.

**Figure 1:**
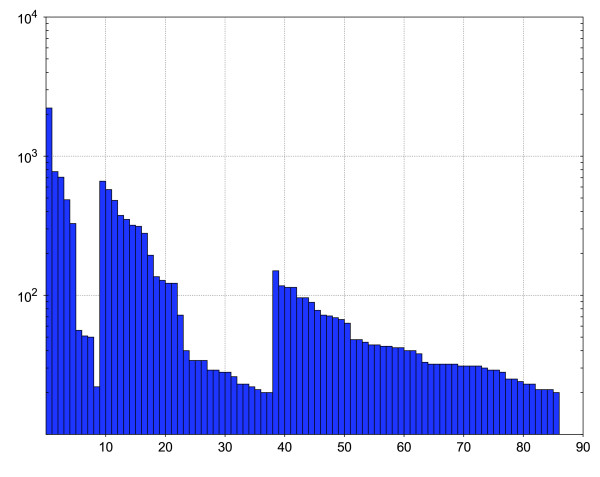
**Size distribution in MDR families**. The bar chart shows the number of seed sequences for the 86 stable and reliable HMMs using inclusion control strategy II, ordered by MDR family number. The number of seed sequences varies from 20 to 2217, and the average is around 137 sequences. The other strategies produce very similar size distributions.

As can be seen in Figure 1, the number of members varies from 20 to 2217 among these HMMs, with an average number around 135 sequences. The average sequence identity within the individual families varies from 39.1% to 93.7%, with an average-of-averages of 61% and a sample standard deviation of 11.3 percent units. The largest MDR family is that of ADH (MDR001) with presently well over 2000 members. Among the top 10 in size we also find PTGR (MDR002), FAS (MDR003), CAD (MDR010), QOR (MDR011), QORX (MDR004), YHDH (MDR012), FDH (MDR013), TDH (MDR014) and PDH (MDR005). Numerical details on the individual MDR families are available in Table 1, showing also the kingdom representation for the families. Further details on conservation and cofactor preference are available in Additional file 1: mdr-properties. The HMM database is available as Additional file 2: mdr-pfam and the accompanying sequence data is available in Additional file 3: mdr-sequences. Coloured MSAs for the families are shown in Additional file 4: mdr-figures.

#### Dendrogram

**Figure 2:**
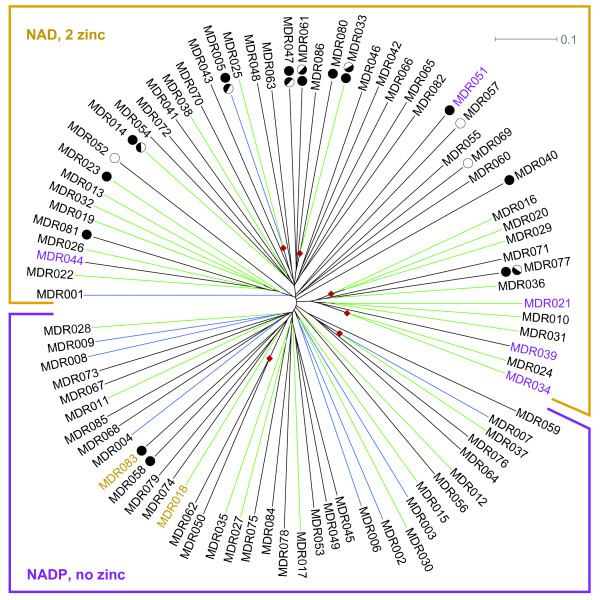
**Dendrogram of the 86 MDR families**. A ClustalW neighbour-joining dendrogram of representative sequences from the 86 MDR families. Blue lines indicate families with at least one human member and green lines indicate families with at least one eukaryotic member. The families with members that bind NAD and two Zn^2+ ^are generally found in the upper half of the dendrogram (indicated with a yellow frame) while those with members that bind NADP and no Zn^2+ ^are almost exclusively found in the lower half (indicated with a purple frame). Exceptions to the NAD/NADP cofactor preference indicated within the frames are highlighted by labels in the opposing colour. Exceptions to the number of bound Zn^2+ ^indicated within the frames are shown using bullet symbols. Two filled bullets correspond to 2 Zn^2+^, one filled bullet to 1 Zn^2+^, and an unfilled bullet to 0 Zn^2+^. Half-bullet symbols are used to indicate cases where the ligands for one of the Zn^2+ ^are conserved only among part of the family members. Although bootstrap values are consistently low (not shown), six branch points were observed in over 900 of the 1000 bootstrap reconstructions of the dendrogram (indicated using red diamonds).

In order to investigate the interrelations between these families we built a bootstrapped ClustalW neighbour-joining dendrogram of representative sequences (Figure 2). The families are approximately equidistantly positioned in the tree and the bootstrap values (not shown) are consistently low. The topology of the MDR family tree is thus similar to that of its sister protein superfamily, SDR. However we do see some trends in the groupings, as families that bind NAD and two Zn^2+ ^are generally found in the upper half of the dendrogram (largely eukaryotes) and those that bind NADP and no Zn^2+ ^are generally found in the lower half of the dendrogram (largely prokaryotes).

Some families with similar function tend to cluster consistently in the dendrogram (shown using red diamonds). For example, SDH (MDR005; sorbitol, mannitol and polyol DHs) is found together with yPDH (MDR025; yeast polyol DHs) in 996 out of 1000 bootstrap reconstructions of the dendrogram. Similarly, with a bootstrap value of 949 BDH1 (MDR033; eukaryotic butanediol DH) clusters with its bacterial sibling family bPDH (MDR080), and in all bootstrap reconstructions of the dendrogram the prokaryotic crotonyl CoA reductases in CCR and CCR2 cluster (MDR050 and MDR062).

Furthermore, a few families that were previously treated as a single family [1] are now represented by two or more HMMs, that cluster together in a significant number of bootstrap reconstructions. For example the tetrameric ADHs of MDR016 (bADH, bacterial ADHs), MDR020 (yADH, yeast ADHs) and MDR029 (yADH2, yeast ADH2) are found together in 924 cases, and were indeed previously described as a single evolutionary group under the name TADH. Also, the mitochondrial trans-2-enoyl-CoA reductases of MDR007 and MDR037 (previously described as MECR) are found together in 987 cases.

Lastly, MDR024 and MDR034 cluster together in all bootstraps reconstructions of the tree. However, not much is known about these families. They share internal pairwise sequence identities of over 50%, have eukaryotic and prokaryotic members, and apparently bind two Zn^2+ ^ions.

#### Correlation with known families

In a recent MDR review [1], we highlighted 17 families of interest, based on family size or presence of a human member sequence. That selection of families was manual, as was the refinement of their derived HMMs. Using the current more systematic approach to subclassification we see that almost all of the families highlighted in the review still have well-correlating counterparts, the exceptions being the smallest families DOIAD, QORL and RT4I (cf. Table 2). Presumably, there is still not enough data available on these families for meeting the minimum size requirement employed in this study. Notably, the CAD, TAD and MECR families were now represented by two or more HMMs (see below). As a check for consistency, we therefore also produced merged HMMs for these families. All of these produced non-overlapping HMMs that became stable in the first or second iteration, demonstrating our algorithm's capability of finding evolutionary supersets for coherent and related subsets. Conversely, the MCAS and ACR families are now incorporated into a single, much larger HMM. This may be desirable from a functional perspective since both MCAS and ACR have functions related to fatty acid synthesis. Knowledge on these families have now grown to roughly twice that at the time of our review, with the exception of DOIAD, QORL, RT4I (the three smallest families mentioned above), and QOR (fifth smallest family).

**Table 2:** Correlation with known families

Family	Number	Size
ADH	MDR001	931 → 2217
CAD	MDR010, MDR021	520 → 661, 122
LTD	MDR002	427 → 774
TADH	MDR016, MDR020, MDR029	330 → 313, 128, 29
YHDH	MDR012	295 → 481
BPDH	MDR011	229 → 575
PDH	MDR005	218 → 328
TDH	MDR014	215 → 351
BurkDH	MDR041	67 → 114
MCAS, ACR	MDR003	58, 25 → 706
MECR	MDR007, MDR037	49 → 51, 20
VAT1	MDR008	39 → 50
QOR	MDR009	28 → 22
DOIAD	-	-
QORL	-	-
RT4I	-	-

MDR families by name used in [1]. The CAD, TADH and MECR families are now represented by two or three HMMs, and the MCAS and ACR families are now incorporated into a single, much larger HMM. The DOIAD, QORL and RT4I1 families are not included in this study, because the amount of data available on these families is insufficient to satisfy the minimum inclusion size employed here.

#### Zn^2+ ^content

The MDR members have 0, 1 or 2 Zn^2+ ^ions per subunit. One Zn^2+ ^ion is located at the catalytic site and is designated catalytic Zn^2+^, while one Zn^2+ ^ion is stabilising a loop encompassing residues 94-117 (human ADH1beta numbering) and is designated structural Zn^2+^. Of the 86 MDR families now characterised, we find that 35 of the MDR families seemingly have 2 Zn^2+^, i.e. both the catalytic and structural Zn^2+^, 7 MDR families have 1 Zn^2+^, while 38 MDR families seem to have no Zn^2+^. Among Bacteria, over half (54%, Table 3) are MDR forms without Zn^2+^, while among Eukaryota, 66% of the MDR forms have 2 Zn^2+^. This finding indicates that the Zn^2+ ^have been recruited early but not initially upon the MDR superfamily formation.

**Table 3:** MDR forms with 2 Zn^2+ ^and no Zn^2+^

	0 Zn^2+^	2 Zn^2+^
Archaea	5	28
Bacteria	3907	3395
Eukaryota	1041	1994

The numbers show number of MDR forms with no Zn^2+ ^and with 2 Zn^2+ ^in each kingdom.

Six MDR families (MDR005, MDR014, MDR033, MDR047, MDR061 and MDR077) consist of members where some have 2 Zn^2+ ^while other members only have 1 Zn^2+^, where typically one or more of the ligands for the structural Zn^2+ ^have been lost. This variable Zn^2+ ^content within the same MDR family has not been described before. Furthermore, in two MDR families (MDR058 and MDR083) the members seem to retain the structural Zn^2+ ^but not the catalytic Zn^2+^, which is clearly different from the typical pattern within the MDR superfamily. This also applies to part of the family MDR033 (cf. above).

#### Coenzyme preference

We have also looked at the coenzyme specificity as judged from available data or the presence of NAD- or NADP-specific ligands. A total of 44 families use NAD as cofactor, while 40 families use NADP. In nearly all families the coenzyme preference seems to be constant for all members, but in one family (MDR023, cf. below) half of the members bind NAD (36/72), while the other half prefers NADP.

#### Correlation between Zn^2+ ^content and coenzyme preference

With many MDR families now characterised, we can distinguish novel patterns. There is a clear correlation between Zn^2+ ^content and coenzyme preference. Most of the MDR families with 2 Zn^2+ ^prefer NAD as cofactor and are thereby generally acting as dehydrogenases. Of the 35 families with 2 Zn^2+^, 30 exhibit preference for NAD. Similarly, there is a correlation between no Zn^2+ ^content and utilisation of NADP as cofactor. Of the 38 families with no Zn^2+^, 34 exhibit preference for NADP and are consequently largely reductases.

As exceptions from these general patterns we find four families, YJGB (MDR021), bADH4 (MDR039), MDR044, and MDR034 of yeast and bacterial alcohol dehydrogenases, which all appear to bind NADP despite having both the structural and catalytic Zn^2+ ^cofactor. Further exceptions are the four families MDR018, YCJQ (MDR052), BCHC of the bacterial chlorophyll synthesis pathway (MDR057), and MDR069, which appear to prefer NAD despite lacking the structural and catalytic Zn^2+ ^cofactors.

#### Species distribution

**Figure 3:**
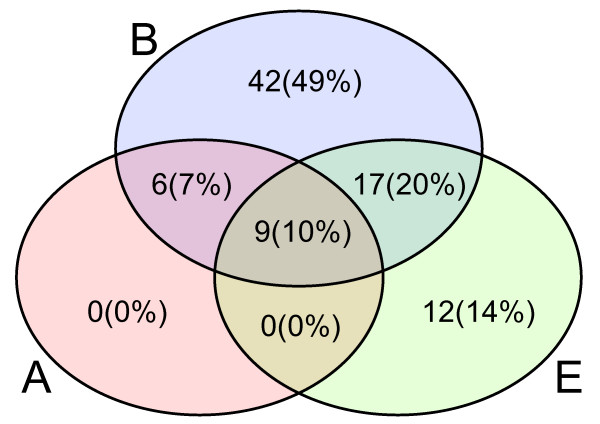
**Venn diagram of kingdom representation in MDR families**. This diagram illustrates the presence of sequences from the different kingdoms in the MDR families with number (and proportion). Each circle encapsulates those families that have at least one sequence from that corresponding kingdom. Blue (top) denotes bacteria, red (left) denotes archaea, and green (right) denotes eukarya. Intersections encapsulate families having at least one member sequence from the corresponding kingdoms.

**Figure 4:**
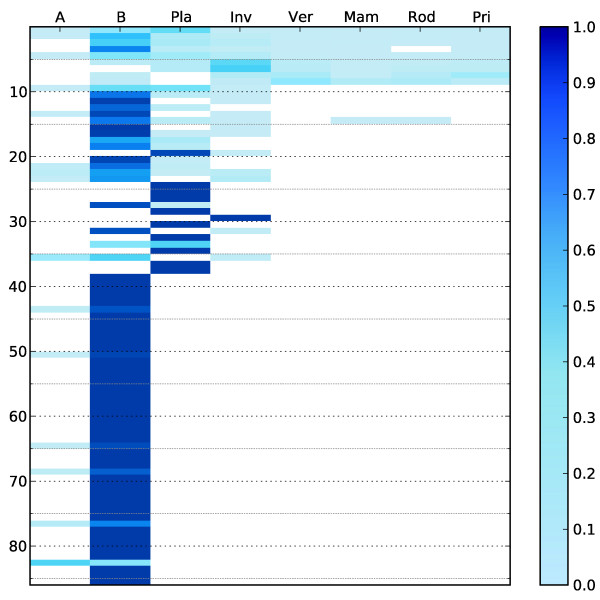
**Species distribution in MDR families**. The species distribution in the individual families is shown using a gradient from white to dark blue (0% to 100% of the family). The columns represent the species groups A - archaea, B - bacteria, Pla - plants, Inv - invertebrates, Ver - vertebrates, Mam - mammals, Rod - rodents, Pri - primates. The families are ordered according to the MDR family enumeration. The numerical values underlying this figure are available in Additional file 5: mdr-distribution.

As shown in Figure 3, nearly 50% of the families are purely bacterial, while only 14% are purely eukaryotic. None is purely archaeal, but 17% of the families are represented in Archaea. 10% of the families have representatives from all kingdoms of life. Figure 4 shows more details on the species distributions in the families (cf. Additional file 5: mdr-distribution for exact figures). Still, most families are dominated by prokaryotic species, although there are some families with only plant sequences, and some with sequences from other eukaryotes, for example MDR030 (dFAS) that consists of 28 sequences exclusively from the invertebrate *Dictyostelium discoideum*. The seed sequences for this HMM have 57.5% average identity.

#### Conserved charge differences

Several MDR families are found to have a large number of strictly conserved charged residues causing a large net charge, which might indicate special binding properties or interaction with strongly charged substrates. The BurkDH (MDR041) has a conserved net charge of -7, while the YJDL (MDR054) and MDR070 both have a conserved net charge of -5. Furthermore, BDH (MDR033), MycDH (MDR086) and MDR038 also have a conserved net charge of -5 (not including Zn^2+ ^ligand glutamate residues). When including non-conserved charges, the numbers become even more pronounced for the first five families (average net charges -8.6, -7.2, -11.1, -9.3 and -11.0, respectively) while only MDR038 has balancing non-conserved charges (average net charge -3.6). All these six families can be expected to have a positively charged substrate or interact with a positively charged protein.

MDR037 is the only family found to have a large positive conserved net charge (+11). However these charges are partially balanced by non-conserved positive charges, leaving an average net charge of +8.7. This family could consequently be expected to interact with negatively charged substrates or proteins.

#### Differences in conservation between the catalytic and coenzyme-binding domains

Looking at the number of conserved residues in the two domains, measured as those attaining a cscore over 95% (conservation score [22]), we find that some MDR families show noticeable differences between the two domains. A total of 14 MDR families were found with more than twice as many conserved residues in one domain compared to the other. There are 10 MDR families with twice as many conserved residues in the catalytic domain as in the coenzyme-binding (Table 4), of which the most extreme cases are SDH (MDR005) with a ratio of 3.5, and MDR013, MDR070, and MDR080, all with a ratio of about 3.0. There are 4 MDR families with twice as many conserved residues in the coenzyme-binding domain as in the catalytic one (Table 4), of which the most extreme cases are VAT1 (MDR008) with 3.7 times difference and MDR035 with 2.25 times difference. These differences might reflect differences in functional and/or structural properties.

**Table 4:** Domain conservation ratios.

Family	Name	Catalytic	Coenzyme	Ratio
MDR005	PDH	28	8	3.50
MDR080	bPDH	97	32	3.03
MDR070		30	10	3.00
MDR013	FDH	39	13	3.00
MDR047		47	20	2.35
MDR075		65	28	2.32
MDR032	bADH2	60	27	2.22
MDR065		41	19	2.16
MDR076		43	20	2.15
MDR004	QORX	17	8	2.13

MDR002	PTGR	7	14	0.50
MDR035		12	27	0.44
MDR003	FAS	8	18	0.44
MDR008	VAT1	10	37	0.27

This table shows the number of strictly conserved positions in the catalytic and coenzyme binding domains, respectively. Furthermore, the ratio between these numbers is calculated. The table only lists families with a ratio of 2 or more and of 0.5 or less.

### Selected MDR families

The largest families and those with known functional data of specific interest are described below.

#### The 10 largest families

Notably, all of the 10 largest MDR families have a high degree of conservation in just one of the two domains (five have over twofold conservation difference between domains, Table 4).

**1. MDR001 - ADH **This family contains the classical alcohol dehydrogenases, including human class I-V alcohol dehydrogenase. Knowledge of this family has grown from 931 members to 2217 since our last review. It is currently the largest MDR family, and 116 of its members are found in the Swiss-Prot database. 59% of the members are eukaryotic and the average amount of pairwise sequence identities is high but variable (51% with 19 percent units standard deviation). The members of this family have both the structural and catalytic Zn^2+^, in agreement with their NAD preference. The catalytic domain conservation is close to twice (89%) that of the cofactor binding domain (17 versus 9 positions with cscores over 95%), indicating that the catalytic machinery is well-conserved through this enzyme family.

**2. MDR002 - PTGR **This family of NADP binding prostaglandin reductases was previously described as LTD and the number of known members has increased from 427 to 774, with only 17 in the Swiss-Prot database. While the family contains human prostaglandin reductases 1 and 2, just over 2/3 of the members are prokaryotic, and its average pairwise sequence identity is only 42%. Most likely, the prokaryotic members have other substrates since prostaglandins are only characterised in verterbrates. Additionally, their conservation is much higher in the cofactor binding domain than in the catalytic domain (14 *versus *7 positions with cscores above 95%), further supporting that the substrate spectrum might vary between the members. This family binds NADP and no Zn^2+^, in agreement with the reductase function.

**3. MDR003 - FAS **This family of multidomain fatty acid synthases was previously described in part as two separate subfamilies ACR and MCAS, but is now represented by a single HMM with 706 members instead of the previously found 25 and 58 members, respectively. Members are prokaryotic to 59% and the average sequence identity is only 39%. Also in this family conservation is much higher in the cofactor binding domain than in the catalytic domain (18 versus 8 positions with cscores above 95%). The MDR domains in these enzymes are annotated with enoyl reductase functionality (by similarity) in Swiss-Prot. A reductase function would be in accordance with their apparent NADP-preference and lack of conserved Zn^2+ ^ligands.

**4. MDR010 - CAD **This family with cinnamyl alcohol DHs, mannitol DHs and sinapyl DHs has both eukaryotic and prokaryotic sequences. It was previously classified together with members from MDR021 as one family [1], but is now represented by two disjoint HMMs, where the latter one is smaller and covers mostly uncharacterised bacterial sequences (cf. Table 2). MDR010 now has 661 known members, of which 32 can be found in the Swiss-Prot database. The catalytic domain is more well-conserved than the cofactor binding domain (with 79% more positions in the former domain attaining cscores over 95%). The members of this family bind NAD and two Zn^2+^.

**5. MDR011 - bpQOR **This family of mainly yeast and bacterial quinone oxidoreductases was previously described under the tentative name of BPDH (for Bacterial and Plant DHs). Now however, as the number of known member sequences has grown from 229 to 575, it also includes six sequences from *Neurospora*, *Leishmania*, *Trypanosoma *and *Dictyostelium *(cf. Additional file 3: mdr-sequences). Still, only five of the sequences are currently in Swiss-Prot.

**6. MDR004 - QORX **This family encompasses putative quinone oxidoreductases from many species, including human quinone oxidoreductase PIG3 (tumour protein p53 inducible protein 3), which is the only Swiss-Prot sequence among its 486 members. The rate of conservation is over twice as high in the catalytic domain as in the cofactor binding domain. Substrate specificity is still unknown. The PIG3 is strongly expressed in neuronal cells of cerebral cortex and in respiratory epithelial cells of nasopharynx [23].

**7. MDR012 - YHDH **This family was previously described as a bacterial family under the name YHDH. It has since grown from 295 to 481 members, including a single eukaryotic sequence from the tunicate *Oikopleura dioica *[UniProtKB:Q66S07].

**8. MDR013 - FDH **This family contains 375 members including S-(hydroxymethyl)glutathione dehydrogenase from *Methylobacter marinus*. 4 of its members are from the Swiss-Prot database. It has an average pairwise sequence identity around 47%, and much of the conservation is seen in the catalytic domain, which has three times as many positions with cscores over 95% than does the cofactor binding domain (39 versus 13 positions).

**9. MDR014 - TDH **This family of threonine dehydrogenases has been previously reviewed, for example in [1], and currently has 351 members. They have conserved ligands for both Zn^2+ ^cofactors, with the exception of 18 rhodobacteral and 6 other bacterial sequences that do not have the sequence motif that binds the structural Zn^2+^.

**10. MDR005 - PDH **This family of sorbitol/xylitol DHs and D-xylulose reductases now has 328 members. Most of them bind 2 Zn^2+^. However, in 113 of its members the structural Zn^2+ ^binding loop is seemingly retained, although the Zn^2+ ^ligands themselves are not conserved. Notably, although this family contains both dehydrogenases and reductases, structural Zn^2+ ^ligand retention is not correlated with enzymatic function. For example, the structural Zn^2+ ^ligands are present in sorbitol DH in fission yeast but not in baker's yeast, and nor are they present in the latter's D-xylulose reductase.

Furthermore, 28 positions in the catalytic domain attain a cscore over 95%, which is 3.5 times the number of such positions in the cofactor binding domain, indicating evolutionary constraints on the catalytic residues.

**Other families of interest **MDR009 - vertQOR - is a family dominated by quinone oxidoreductases and zeta-crystallins from vertebrates, where the single non-eukaryotic sequence is from the bacterium *Geobacter uraniireducens strain Rf4*. It thus contains both enzymes and structural proteins of the lens, which are recruited to that function [24]. The family also includes one lancelet form from *Branchiostoma floridae *[UniProtKB:B6NHZ5] which was deleted in UniProtKB release 15.8 since the corresponding gene was not present in the latest assembly of the genome. However, BLAST searches against the UniProtKB and NCBI nr [25] databases reveal that the most similar known sequence is from platypus and shares only 60% identities with this deleted form. The most similar non-vertebrate form is from *Monosiga brevicollis *(a choanoflagellate) at 53% identities, and is superseded by 33 more similar vertebrate sequences. It is therefore possible that this deleted lancelet form is not an artifact originating from a contaminating sequence, but may reappear in a later release of the *Branchiostoma floridae *genome.

In the MDR019 family, eight yeast sequences of the 136 members seem to bind NADP instead of NAD. Four of these sequences are from species having two forms of this protein, of which one has NAD-typical ligands and the other NADP-typical ones. The MDR023 family of ADHs from eukaryotic parasites as well as thermophiles and other prokarya consists of two subgroups, one apparently using NADP as cofactor, the other NAD. This difference does not appear to stem from species multiplicity, as all sequences from any single species exhibit the same cofactor binding motifs. A small subset of four sequences show intermediate properties.

The MDR020 (yADH) family contains tetrameric ADHs from various yeasts, and also two forms from *Caenorhabditis elegans *and one from *Caenorhabditis briggsae*.

MDR022 (giFDH) contains glutathione-independent formaldehyde dehydrogenases from bacteria and yeast. Family MDR030 (dFAS) has 28 members, all uncharacterised polyketide synthases from the slime mold *Dictyostelium discoideum*. Yet the average pairwise sequence identity within the family is merely 57.5%. The MDR033 family consists of yeast (R,R)-butanediol dehydrogenases. Here, 10 of 23 members have a glutamate to glutamine substitution for the third catalytic Zn^2+ ^ligand, which is located in the first transdomain alpha helix. This substitution should preclude binding of the catalytic Zn^2+^. However, the representative from *Saccharomyces cerevisiae *[Swiss-Prot:P39713] is annotated as having the catalytic Zn^2+^, despite having only two ligands. However, all of these 10 sequences come from species that have two forms of this protein, and where the sibling sequences have an intact third ligand for the catalytic Zn^2+^. Furthermore, this family has an unusual insertion in the structural Zn^2+ ^loop, between the first and second Zn^2+ ^ligands. The length of this insertion seems to vary between members. Also, as noted above, this family has a significant conserved net charge of -5, or -8.6 when including non-conserved charges (excluding Zn^2+ ^ligand glutamate), and could therefore be expected to have positively charged substrates or interaction partners.

In the MDR047 family of mainly bacterial starvation-sensing oxidoreductases, 10 out of 72 members have lost the ligands for the structural Zn^2+ ^cofactor through a deletion precisely at the binding motif in the Zn^2+ ^binding loop. The catalytic domain has a higher degree of conservation with 47 positions (excluding structural Zn^2+ ^ligands) attaining a cscore over 95%, which is 2.35 times the number of such positions in the cofactor binding domain.

The members of the bacterial family MDR058 seem to have ligands for the structural Zn^2+ ^cofactor, but remarkably not for the catalytic Zn^2+ ^cofactor. The members also have a conserved proline-rich motif near the N-terminus, the most pronounced being PPPPPPGP at position 18 in a sequence from *Aurantimonas *sp. SI85-9A1 [UniProtKB:Q1YMF6]. Also the bacterial family MDR083 presents the uncommon feature of having ligands for the structural but not the catalytic Zn^2+^.

Only 28 out of 40 members of the bBDH (MDR061) family of bacterial 2,3-butanediol dehydrogenases have ligands for the structural Zn^2+ ^cofactor. The remaining 12 still have a loop that is four residues shorter than its Zn^2+ ^binding counterpart. Similarly, in the family MDR077, only 18 out of 28 members have ligands for the structural Zn^2+ ^cofactor. The remaining 10 have seemingly lost the Zn^2+ ^binding motif though consecutive substitutions, as the degree of similarity to their Zn^2+ ^binding siblings is still high in all other positions in the loop.

The MDR069 family is unusual in that it appears to bind NADP despite having both the structural and catalytic Zn^2+ ^cofactor. The conservation is low in the C-terminal part of the catalytic domain, and the usually well-conserved positive charge near the terminus is absent. There is a highly positive conserved motif near the NAD-binding VGV motif in 1PL8, as represented by RKRGR in a *Ralstonia solanacearum *sequence [UniProtKB:A3RXV2].

The family MDR070 has an unusual, poorly conserved region of variable length, close to the positive NADP ligand 24 residues C-terminally of the glycine-rich NADP binding motif (GxGxxG). As an effect, the number of positions attaining cscores over 95% in the catalytic domain is threefold that of the cofactor binding domain, with only 10 such positions. Furthermore, proteins of this family have a conserved net charge of -5, and may thus have have positively charged substrates or interaction partners.

### The algorithm

Searching UniProtKB we find sequences for 15136 MDR domain pairs in 14996 proteins. Clustering sequences with more than 40% pairwise identities leaves 1032 clusters, where known families such as ADH, PDH and PTGR are uniquely represented. 146 of these clusters consist of more than 20 sequences, and were used for protein family HMM generation, making a grand total of 10193 sequences.

Some proteins, mainly polyketide synthases and type I fatty acid synthases like [UniProtKB:Q5CQX7] and [UniProtKB:O96554] from *Cryptosporidium parvum*, contained several MDR domain pairs that were sufficiently divergent to fall into separate clusters under the chosen clustering rule. No special regard was given to these clusters during HMM construction, refinement or inclusion control, however all of them eventually converged on (subsets of) the same evolutionary group, namely MDR003 - FAS.

After 4 reiterations all HMM refinements using inclusion control strategy II were stable (our favoured strategy, see below). After resolving overlaps we obtained stable HMMs for 86 MDR families. The corresponding 11579 seed sequences provide just over 76% coverage of the MDR superfamily (cf Table 5). Two out of these 86 stable HMMs (MDR035 and MDR086) retained too few sequences for continued refinement after the first inclusion control step (cf. Methods below). These HMMs comprise a total of 42 seed sequences and are stable, and were thus still kept (but explicitly annotated as potentially less reliable). Past the first inclusion control step, it was possible to classify all remaining HMMs as reliable in all of the strategies.

**Table 5:** Characteristics for different inclusion control strategies.

Strategy	Families	Sequences	Reiterations	Subsets
I, exclusive	92 (15)	10401	6	22 {16}
II, intermediate	86 (2)	11579	4	34 {14}
III, inclusive	85 (2)	11657	2	36 {15}

Three different strategies for inclusion control were employed, affecting the number of resulting HMMs as well as their composition and relations. Roman numerals denote the different strategies, in increasing order of inclusiveness. Parenthesised numbers show the number of HMMs that were not affixed with the "reliable" qualifier, due to having too few non-spurious sequences in their seed sets. Numbers in braces denote the number of families having such subsets.

In strategy I, all seed sequences failing the leave-one-out check were excluded. In strategy II, only seed sequences with domain scores lower than noise level were excluded. Additionally, for a left-out seed sequence to be excluded in strategy III, its domain score must fall below 90% of the lowest domain score among the remaining seed sequences.

34 of the refinement processes stabilised on distinct subgroups to 14 of these 84 families, and these were retained separately because of their potential utility for subclassification. It should however be noted that this work does not attempt to provide a comprehensive listing of all MDR subfamilies, but these 86 HMMs are rather presented as a means to aid automated classification.

#### Inclusion control and overlap resolution

In order to ensure consistency in our refined HMMs we evaluated three different leave-one-out validation strategies for seed sequences (cf. Table 5). Unsurprisingly, the most inclusive strategy (III) yielded the most numerous and largest overlaps between seed sets, while exactly the opposite was true for the most exclusive strategy (I). However, for strategy III, the overlaps were generally trivially resolvable, most often simply by raising the inclusion threshold slightly for one of the participants, while for strategy I some overlaps were only resolvable through mergers and subsequent refinement reiterations. As an example; in strategy I, one cluster of 1390 sequences was found to be a subset to MDR001 (1631 sequences) with the exception of 7 sequences which were included late in the refinement process. A neighbour joining dendrogram of the smaller cluster showed that these sequences originated from separate subgroupings, and although it was possible to exclude them from the cluster by removing the 70 lowest scoring seed sequences, these 7 sequences would be re-included in the subsequent refinement step. Conversely, merging the 7 sequences into the larger set yielded a HMM that became divergent in the subsequent refinement step. In the more inclusive strategies on the other hand, these clusters had a clear subset relation, lending further incentive to favour one of these strategies.

Strategy III ultimately proved to be the fastest, since it required the fewest reiterations of refinement and inclusion control before all HMMs were stable. The apparent speed loss of the more inclusive strategies impacted by the time consuming database searches during inclusion control was more than compensated by the ease of overlap resolution and the lower number of refinement reiterations required for convergence. The intermediate strategy (II) was found to be an optimal compromise between I and III, occupying the middle ground in these aspects; it is reasonably fast and offers the ease of overlap resolution of strategy III. Another clear advantage of strategy II is that it does not rely on user input beyond initial HMM creation, but instead purely uses relations in data to produce the resulting models.

One concern during the development of the refinehmm algorithm was that inclusion of partial sequences into the seed sets could lead to attenuation of predictive power of the model, since this would introduce a biased delete state preference near the N- and C-termini of the model. To counteract this we attempted to introduce a minimum model coverage requirement for inclusion of new seed sequences, expressed as a percentage of the model length, and we evaluated the thresholds 90%, 85%, 80% and 0%. Ultimately, the 0% coverage limit proved to be sufficient, as the hmm_ls HMM type itself provides sufficient penalisation of partial sequences to ensure that they will not increasingly dominate the refined seed sets.

#### Computation time and parallelisation

Since the individual refinement processes are independent, this algorithm for HMM refinement is trivially parallelisable. Furthermore, the output files and the variant input files are small. The database files are the only large files needed by the algorithm, and as these are invariant, they can be cached on the compute resource. These traits make refinehmm well suited for burst computing on shared resources as well as distributed resources such as Swegrid (the Swedish computational grid). Indeed, all refinements and leave-one-out comparisons to UniProtKB were computed on Swegrid resources using Biogrid runtime environments (bioinformatics virtual organisation in the Nordic Data Grid Facility) [26]. Internal leave-one-out comparisons were fast enough to compute locally, rather than distributed. The refinehmm algorithm uses version 2.3 of HMMER, but is written so that a transition to HMMER 3.0 will be straightforward once a stable release with glocal alignment capabilities is available. As the HMMER database search is by far the most time consuming step in the refinehmm algorithm, we estimate that transitioning will decrease the total computation time by at least an order of magnitude, and quite possibly even more.

### Validation

The HMMs were built and refined against version 14.8 of UniProtKB, and in order to evaluate the consistency of the annotation of the 86 families defined in this work, we investigated the annotation of all new MDR sequences in new releases of Swiss-Prot up to version 15.10. We found 118 new matches against the PfamA MDR HMMs, out of which 90 were upgrades from UniProtKB/TrEMBL sequences, and 28 were novel entries in Swiss-Prot. Our models detected all of these new sequences (except for 14 of the novel ones, see below) and none of these had annotations that conflicted with our previous observations on their respective families. Out of the 14 sequences that were not detected, 12 were annotated as probable polyketide synthases of *Dictyostelium discoideum*, and would perhaps have been expected to match MDR030 (dFAS). The remaining two ([UniProtKB:Q5AUY5] and [UniProtKB:O42909]) had annotations relating the proteins to ADH-like enzymes, but nothing more functionally specific. Since our models professedly do not provide 100% coverage of the MDR superfamily, it should be expected that some new MDR sequences will not be classifiable through our current library of HMMs. However as more sequence data on MDRs is amassed, further HMMs may be developed using our algorithm, improving the library and its coverage of the MDR superfamily even further.

### Web site

In order to make our results directly useful also for scientists outside the bioinformatics area, we have developed a web site that presents our findings in an easily navigable point-and-click interface. The web site enables users to search and display detailed information about the MDR families, using textual and numeric queries against many of their characterised properties. It also permits online hmmpfam queries against our library of MDR HMMs, making it possible to submit a novel MDR sequence and have it instantly analysed and classified. The web site also provides direct downloads for the underlying MDR HMM library as well as the web site database files, providing the opportunity for advanced users to do more advanced queries offline.

The web site is available at http://mdr-enzymes.org/.

## Conclusions

We have developed an algorithm for HMM refinement that produces stable and reliable HMMs. We have used this algorithm to subdivide a large and complex protein superfamily into smaller units of homologous members, yielding HMMs for 86 MDR families and 32 distinct subsets to 14 of the families. These HMMs are suitable for reliable automated classification of new sequence data. The generated families correlate well with the more empirically chosen family definitions that were employed in our recent review, lending further evidence to the reliability of the algorithm.

Furthermore, 1/4 of the MDR members have only few (or none) close homologues, forming only small families, presently each with less than 20 members. Thus, the MDR superfamily shows a complexity and spread as previously described for the SDR superfamily.

We analysed conservation in these 86 families as well as characterised their NAD(P) and Zn^2+ ^cofactor preference based on mappings to their closest available structures. It was found that MDR families with 2 Zn^2+ ^in general have NAD preference, while those families with no Zn^2+ ^in general had preponderance for NADP.

We have also developed a web site http://mdr-enzymes.org where users can search and display detailed information on the families defined and characterised. It is also possible to scan putative new MDR sequences and have them instantly analysed and classified. These features make our findings directly available and useful also for non-bioinformaticians.

The implementation and support scripts for running the algorithm on computer clusters are available as open source software, and the database files underlying the web site are freely downloadable.

## Methods

### Initial HMM creation

MDR domains were extracted from UniProtKB 14.8 using hmmsearch from the HMMER package [27] with two hmm_ls query HMMs from PfamA; ADH_N [PfamA:PF08240] and ADH_zinc_N [PfamA:PF00107], corresponding to the N-terminal catalytic GroES-like domain and the C-terminal Rossmann fold cofactor binding domain. The extracted domains included linker regions between closely located

ADH_N/ADH_zinc_N match pairs, and singlet matches were expanded to include potentially missed HMM partner matches, simply by moving the corresponding inclusion boundary outwards by the expected length of the linker and absent domain plus a small error margin. Partial sequences shorter than 250 residues were removed before clustering domains with sequence identity higher than 40% using cd-hit [28]. Clusters containing more than 20 sequences were aligned using mafft-L-INS-i [29] and used as seed sets for creating hmm_ls HMMs using hmmbuild from the HMMER package.

### HMM refinement

The refinehmm algorithm is initiated with an HMM which is iteratively refined through progressive database searches and subsequent adaption of the model. In each iteration, all domain matches scoring higher than the worst scoring seed domain are extracted and included in the seed for the next iteration, and when the seed is stable the process is terminated. Refinehmm uses hmmalign and hmmbuild from the HMMER package to build the HMMs; hmmalign to align the seed set for the next iteration against the current HMM, and hmmbuild to produce hmm_ls HMMs from the resulting alignment. The algorithm is implemented in python.

HMMs refinements gathering more than 3000 seed sequences were classed as divergent and aborted, and these were rerun after manual curation of the initial seed sets. For this curation, the HMMs were aligned against their respective seed sets using hmmsearch, and the lowest scoring sequences were progressively removed until HMM refinement became convergent. HMMs that became divergent during later refinement reiterations (see below) were treated identically; by curation of their corresponding initial seed sets and restarting the refinement process from scratch. Finally, the maximum resulting domain score was recorded for each seed sequence, and the minimum of these was recorded as gathering- and trusted cutoffs for each HMM. Noise cutoff was recorded as 0.1 less.

### Inclusion control

All refined (converged) HMMs were subjected to leave-one-out stability checks in order to remove potentially erroneously included seed sequences. Here, the HMM was repetitively rebuilt, leaving out one seed sequence at a time, and in a subsequent database search the domain score of the seed sequence in question and its relation to other domain hits were noted. Three removal strategies were employed (cf. Table 5). In the most exclusive strategy (I), left-out seed sequences were classified as spurious if they received a lower domain score than the remaining seed sequences. In the intermediate strategy (II), the domain score for the left-out sequence must also be exceeded by at least one non-seed sequence in order to be classified as spurious, indicative of a membership strength below the noise level. Additionally, for a seed sequence to be classified as spurious in the most inclusive strategy (III), its assigned domain score must fall below 90% of the lowest domain score among the remaining seed sequences. HMMs having no spurious seed sequences were classified as stable and reliable. Sets with less than 20 non-spurious sequences (MDR035 and MDR086) were classified as stable but potentially less reliable, indicated by an 'x' postfix to the HMM name and with an explicit warning in the HMMs description field. All spurious sequences were then removed from the non-stable seed sets, which were then realigned using mafft-L-INS-i and used to build new hmm_ls HMMs. These resulting HMMs were then re-injected into the refinement process, starting with refinement with refinehmm. Past the first inclusion control step it was possible to classify all remaining refined HMMs as reliable.

For strategy I, inclusion control comparisons were done internally, e.g. against the original seed sets. For the more inclusive strategies, only those comparisons failing the inclusion criteria for strategy I were re-evaluated against the UniProtKB database, greatly reducing the computation time.

### Model overlaps

Overlaps between stable HMMs were resolved in a number of ways. For true subset relations - the most common type of overlap - the superset was kept and the subsets were set aside, giving special note to disjoint subsets given their utility for discrimination between subfamilies.

In partially overlapping sets, the inconsistent domains were typically assigned very low domain scores by one of the HMMs, permitting resolution of the overlap by simply raising the score threshold for the corresponding HMM slightly so that the inconsistent domains - along with a handful similarly low-scoring domains - became excluded. The corresponding seed sets were then edited to reflect this. The rebuilt HMMs were without exception stable and consistent, which was verified by refinehmm.

The few overlaps where the inconsistent domains were assigned high domain scores were instead resolved through mergers, in an effort to find a common evolutionary supergroup. In these mergers, the non-overlapping domains from the smaller seed set were added to the larger seed set, which was then realigned using mafft-L-INS-i and used to build an expanded HMM. The merged HMMs were subjected to refinement using refinehmm in order to ensure stability and consistency.

### Subfamily informatics

Species distribution in each family was determined using the NCBI Taxonomy database [30,31] extended with archaea/bacteria classifications from UniProtKB. One sequence from MDR001 and three sequences from MDR012 belonging to the NCBI Taxonomy "Unclassified" division were disregarded for the species analyses. Sequence identity was calculated as the number of identical residues found by pairwise BLAST [32] divided by the length of the longer sequence. Dendrograms were computed using the ClustalW neighbour joining algorithm [33] applied to mafft-L-INS-i MSAs, and were visualised using Dendroscope [34].

For each family full length sequences were extracted (only the two MDR domains in case of multidomain sequences like the fatty acid synthases), excluding sequences shorter than 270 residues. The sequence sets were redundancy reduced to less than 90% sequence identities using cd-hit, and multiply aligned using mafft-L-INS-i. The best representative structure was determined for each family through blast searches against a database of all MDR structures in PDB [35]. In those cases where the best match yielded an ill-fitting structure due to a highly similar but atypical sequence, we also employed a voting approach that selected the structure that was the best match for the most sequences in the family. The structure sequences were added to the preexisting family MSAs using mafft. Presence of cofactors such as NAD(P), and catalytic and structural Zn^2+^, was assessed through ocular inspection of conserved sequence motifs and conserved ligands in structure. All mapping of motifs and conservation, as well as visualisation of alignments, were made using the msaview multiple sequence alignment analysis package (Hedlund, to be published).

The homogeneity of the families was assessed through comparison of sequence descriptions, giving priority to annotations from the Swiss-Prot database and ignoring descriptions containing qualifiers such as "putative", "uncharacterized", "hypothetical" and "predicted".

## Availability and requirements

The implementation and support scripts for running the algorithm on computer clusters are available as open source software (cf. below, and also Additional file 6: refinehmm and Additional file 7: grid_workdir).

• Project name: RefineHMM

• Project home page: http://sourceforge.net/projects/refinehmm

• Operating systems: Platform independent

• Programming language: Python 2.x (x ≥ 5)

• Other requirements: HMMER 2.x (x ≥ 3)

• License: MIT

• Any restrictions to use by non-academics: License required.

## Abbreviations

ADH: alcohol dehydrogenase; DH: dehydrogenase; HMM: hidden Markov model; MDR: medium-chain dehydrogenase/reductase; MSA: multiple sequence alignment; SDR: short-chain dehydrogenase/reductase; Zn^2+^: zinc ion.

## Authors' contributions

JH has developed the algorithms and software for HMM refinement and executed the experiments. BP conceived and supervised the study. JH, HJ and BP have written the manuscript. All authors read and approved the final manuscript.

## Supplementary Material

Additional file 1 **Further properties of MDR families**. This table extends Table 1 with several additional columns, showing further properties derived during our characterisation of the 86 MDR families; Description (sequence description of one representative seed sequence), Cat 95% (number of positions in the catalytic domain that attain a cscore above 95%), Cat 100% (number of positions in the catalytic domain that attain a cscore of 100%), Cat KR-ED (number of conserved positive charges minus number of conserved negative charges in the catalytic domain; values < -3 or > +1 indicated in yellow), CE 95% (as Cat 95%, but for the coenzyme binding domain), CE 100% (as Cat 100%, but for the coenzyme binding domain), CE KR-ED (as Cat KR-ED, but for the coenzyme binding domain), Cat/CE 95% (Cat 95% divided by CE 95%; values ≤ 0.5 indicated in orange, ≥ 2 in blue), Structure (ID of the PDB structure found to be most similar to any of the sequences in the family), Most similar (ID of the seed sequence most similar to Structure), Zn1cons (presence of catalytic Zn^2+ ^ligands), Zn1charge (sum of charges of catalytic Zn^2+ ^ligands, in the range [-3,0]), Zn2cons (presence of structural Zn^2+ ^ligands), Zn1charge (sum of charges of structural Zn^2+ ^ligands, in the range [-4,0]), Normcharge (sum of conserved charges, excluding conserved Zn^2+ ^ligand charges; values < -4 or > 3 indicated in yellow), Zn (number of Zn^2+ ^cofactors; dual binders indicated in blue, single binders in light blue, and non-homogeneous families are indicated in orange), Zn2 not Zn1 (having structural but not catalytic Zn^2+^; orange if nonzero), 2Zn not NAD (having both Zn^2+ ^ligands without binding NAD; orange if nonzero), 0Zn not NADP (having no Zn^2+ ^ligands without binding NADP; orange if nonzero), NAD (binds NAD), and NADP (binds NADP). Click here for file

Additional file 2 **MDR HMM library**. A database containing the final HMMs for all 86 MDR families described in this work. Compatible with hmmpfam from the HMMER package [27]. Click here for file

Additional file 3 **MDR HMM Sequence data**. Sequence data on the 86 MDR families. The *.seed files contain the sequences that were used to build the HMMs, and *.fasta files contain the corresponding full-length protein sequences. The *.mafft files contain mafft-L-INS-i MSAs for full-length sequences (or member domain pairs in case of multidomain proteins) after redundancy reduction to less than 90% pairwise sequence identities. Click here for file

Additional file 4 **MDR family alignment figures**. Illustrations showing the mafft MSAs from Additional file 3: mdr-sequences. Red sequence identifiers denote sequences from UniProtKB/TrEMBL, while blue correspond to sequences from Swiss-Prot. The position cscores are shown in a bar plot under the MSA. Positions that contain gaps in over 90% of the sequences have been removed from the alignment in order to increase readability. Click here for file

Additional file 5 **Species distribution in MDR families**. The numerical data underlying Figure 4 as a fixed width plain text text file of n(n/N) values where n denotes the number of seed sequences from the evolutionary group in question and N is the size of the corresponding seed set. Click here for file

Additional file 6 **Refinehmm source code**. Python source code for the HMM refinement algorithm described in this work. Requires HMMER 2.x (x ≥ 3) and python 2.y (y ≥ 5) to run. See enclosed README.TXT for information on installation and usage. Click here for file

Additional file 7 **Refinehmm support script source code**. A collection of utilities that facilitate running refinehmm on a computational resource, primarily NDGF Biogrid. Requires Python 2.y (y ≥ 5) and a bash shell to run. See enclosed README.TXT for information on installation and usage. Click here for file
