# Supplementary material for: Subdivision of the MDR superfamily of medium-chain dehydrogenases/reductases through iterative hidden Markov model refinement
Source: BMC Bioinformatics. 2010 Oct 27;11:534. doi: 10.1186/1471-2105-11-534 (PMC2976758; doi:10.1186/1471-2105-11-534)

MDR084

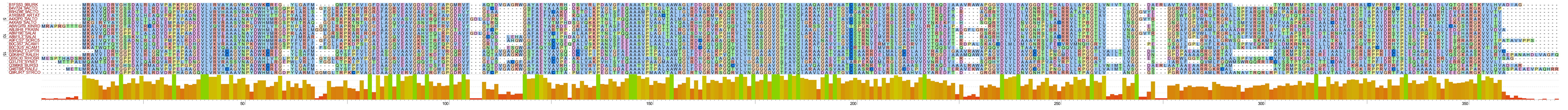

Supplement: Additional file 5 — Species distribution in MDR families. The numerical data underlying Figure 4 as a fixed width plain text text file of n(n/N) values where n denotes the number of seed sequences from the evolutionary group in question and N is the size of the corresponding seed set. [file 1471-2105-11-534-S5.ZIP › mdr/MDR084.pdf]
