# Supplementary figures and images for: Subdivision of the MDR superfamily of medium-chain dehydrogenases/reductases through iterative hidden Markov model refinement
Source: BMC Bioinformatics. 2010 Oct 27;11:534. doi: 10.1186/1471-2105-11-534 (PMC2976758; doi:10.1186/1471-2105-11-534)

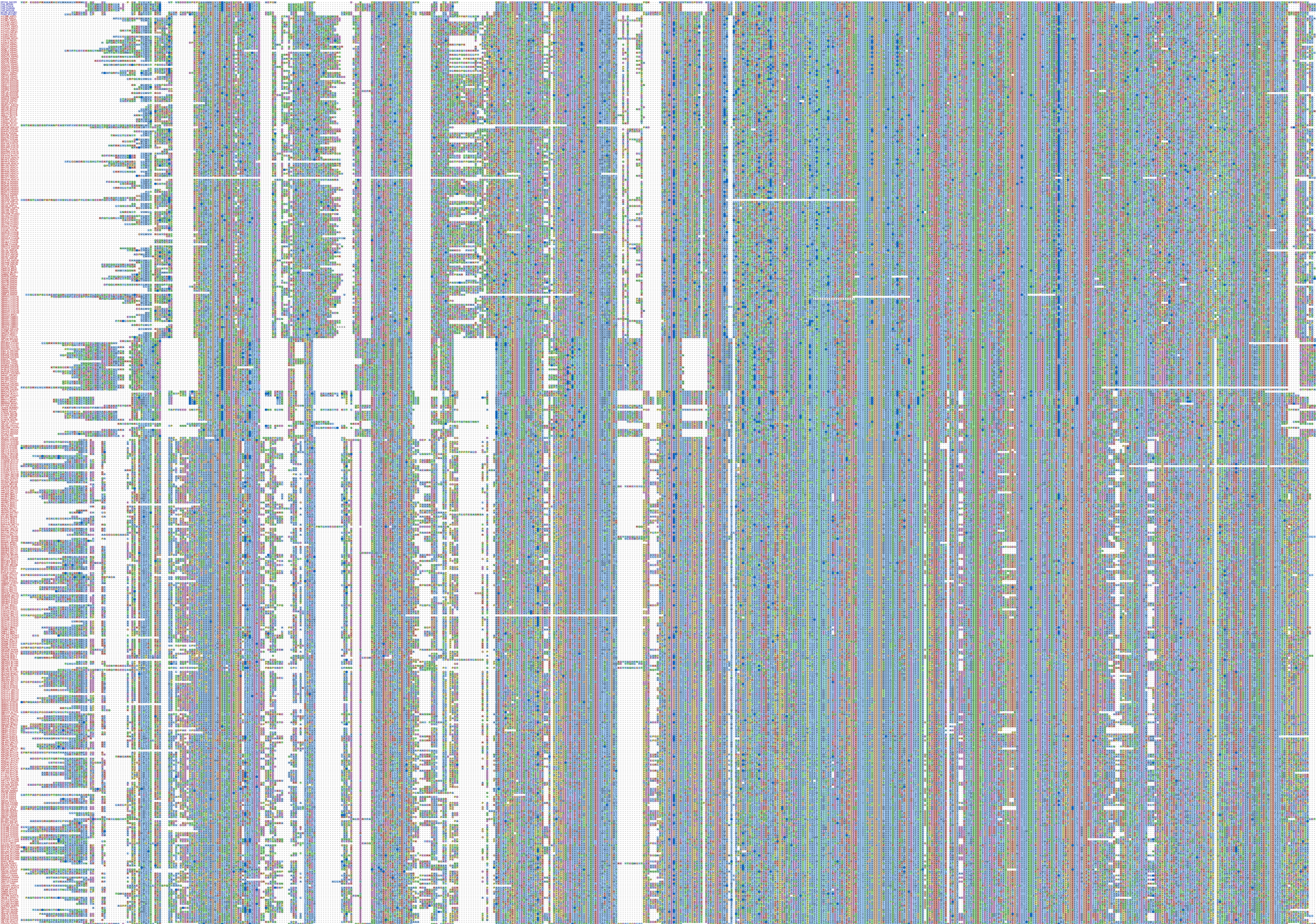

Supplement: Additional file 5 — Species distribution in MDR families. The numerical data underlying Figure 4 as a fixed width plain text text file of n(n/N) values where n denotes the number of seed sequences from the evolutionary group in question and N is the size of the corresponding seed set. [file 1471-2105-11-534-S5.ZIP › mdr/MDR003.pdf]

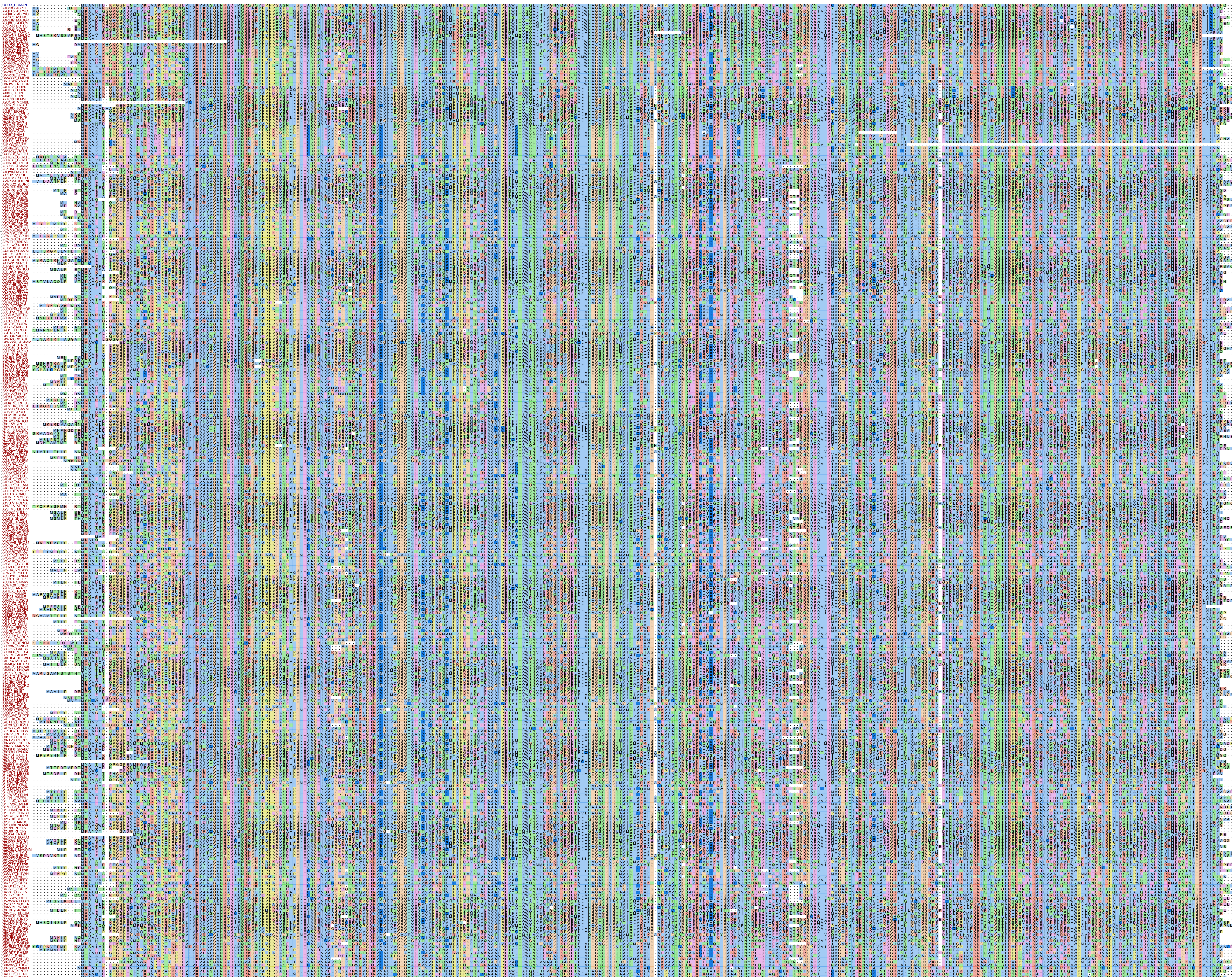

Supplement: Additional file 5 — Species distribution in MDR families. The numerical data underlying Figure 4 as a fixed width plain text text file of n(n/N) values where n denotes the number of seed sequences from the evolutionary group in question and N is the size of the corresponding seed set. [file 1471-2105-11-534-S5.ZIP › mdr/MDR004.pdf]

MDR007

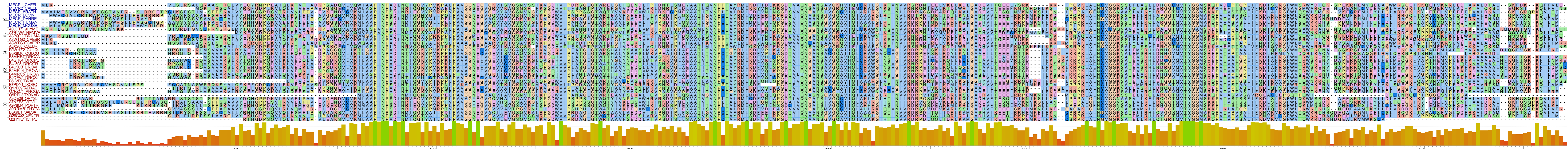

Supplement: Additional file 5 — Species distribution in MDR families. The numerical data underlying Figure 4 as a fixed width plain text text file of n(n/N) values where n denotes the number of seed sequences from the evolutionary group in question and N is the size of the corresponding seed set. [file 1471-2105-11-534-S5.ZIP › mdr/MDR007.pdf]

MDR008

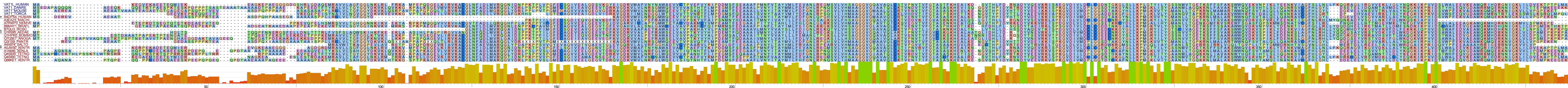

Supplement: Additional file 5 — Species distribution in MDR families. The numerical data underlying Figure 4 as a fixed width plain text text file of n(n/N) values where n denotes the number of seed sequences from the evolutionary group in question and N is the size of the corresponding seed set. [file 1471-2105-11-534-S5.ZIP › mdr/MDR008.pdf]

# MDR009

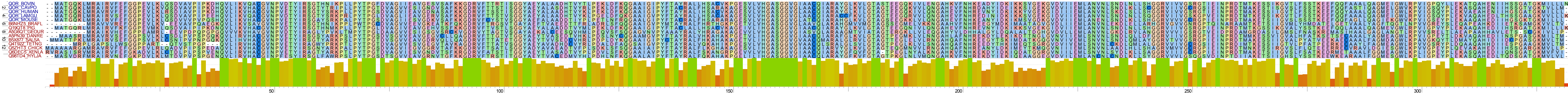

Supplement: Additional file 5 — Species distribution in MDR families. The numerical data underlying Figure 4 as a fixed width plain text text file of n(n/N) values where n denotes the number of seed sequences from the evolutionary group in question and N is the size of the corresponding seed set. [file 1471-2105-11-534-S5.ZIP › mdr/MDR009.pdf]

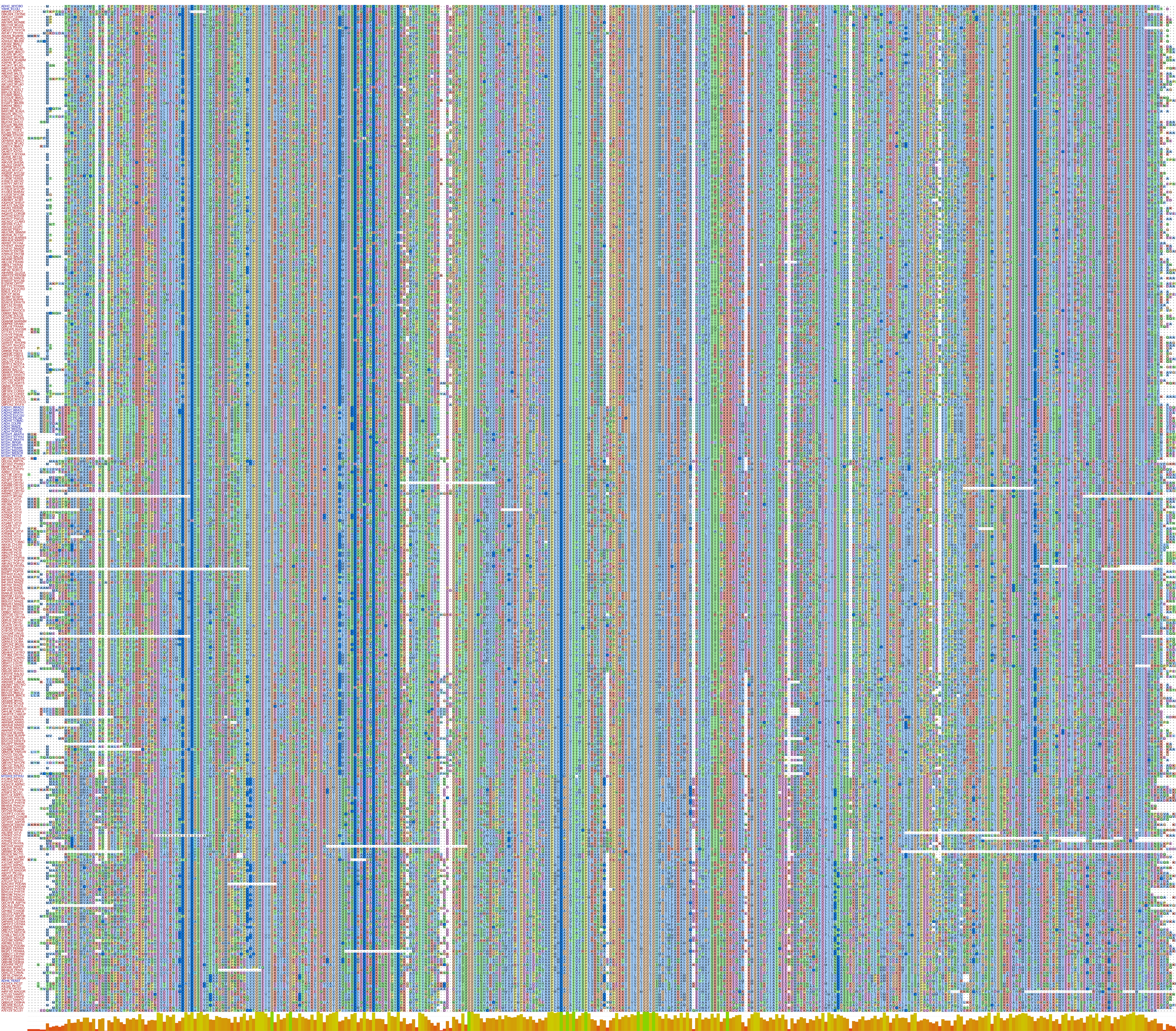

Supplement: Additional file 5 — Species distribution in MDR families. The numerical data underlying Figure 4 as a fixed width plain text text file of n(n/N) values where n denotes the number of seed sequences from the evolutionary group in question and N is the size of the corresponding seed set. [file 1471-2105-11-534-S5.ZIP › mdr/MDR010.pdf]

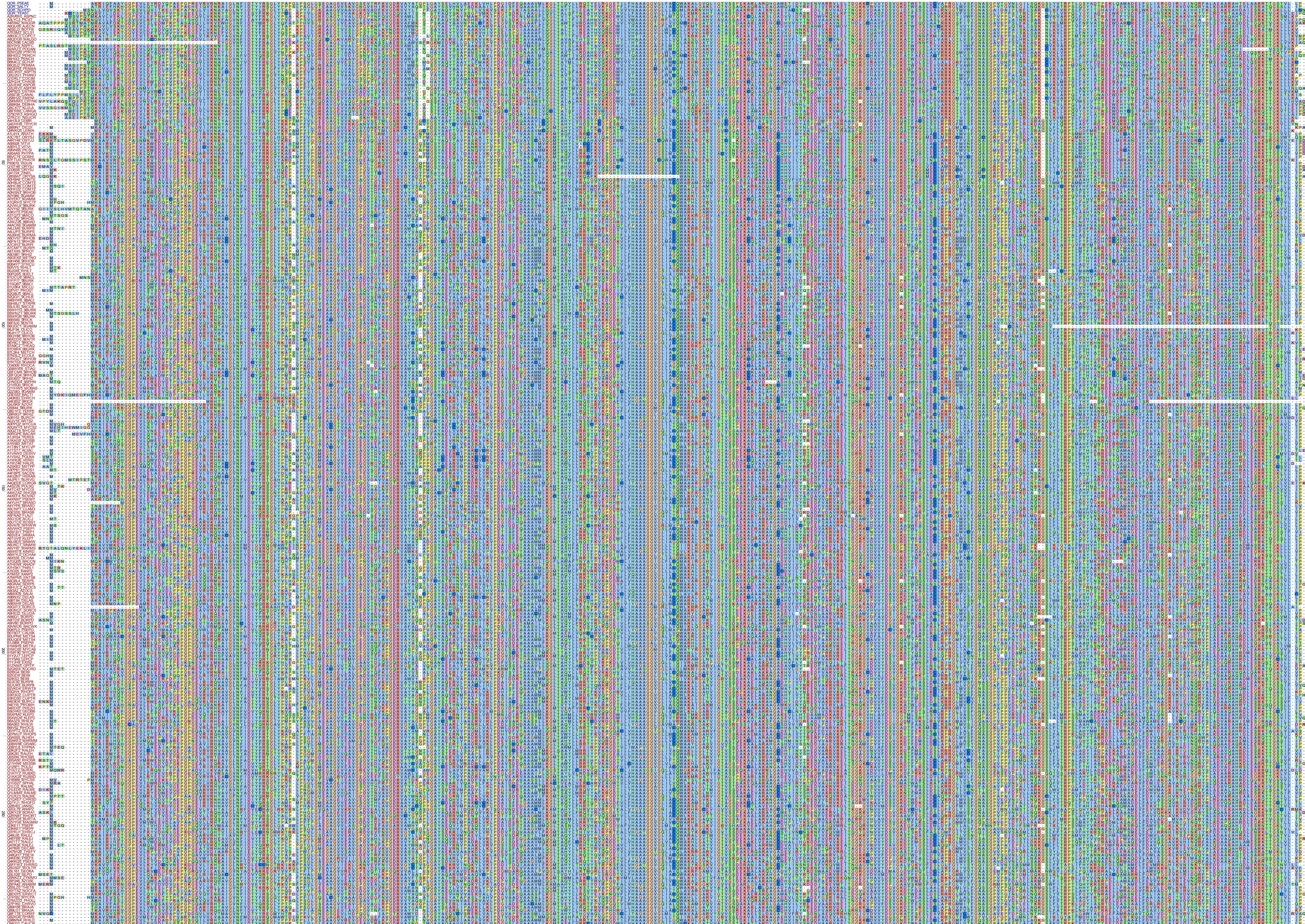

Supplement: Additional file 5 — Species distribution in MDR families. The numerical data underlying Figure 4 as a fixed width plain text text file of n(n/N) values where n denotes the number of seed sequences from the evolutionary group in question and N is the size of the corresponding seed set. [file 1471-2105-11-534-S5.ZIP › mdr/MDR011.pdf]

MDR020

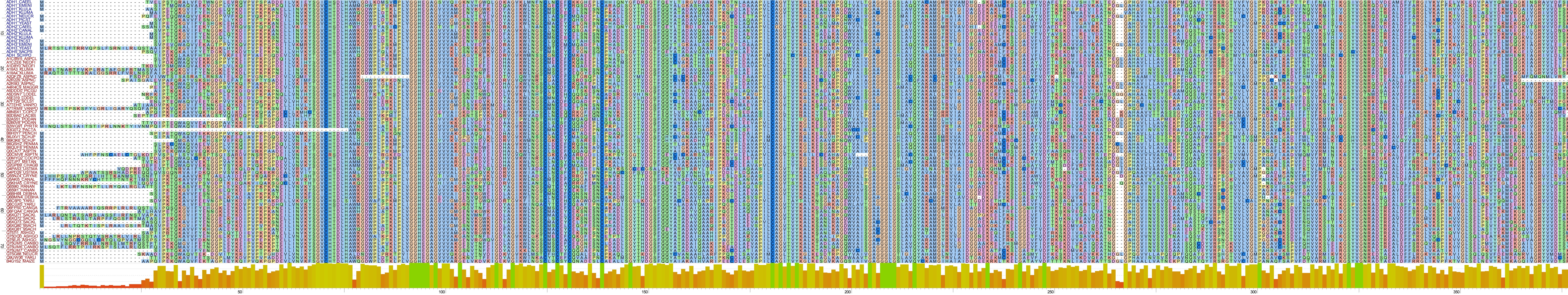

Supplement: Additional file 5 — Species distribution in MDR families. The numerical data underlying Figure 4 as a fixed width plain text text file of n(n/N) values where n denotes the number of seed sequences from the evolutionary group in question and N is the size of the corresponding seed set. [file 1471-2105-11-534-S5.ZIP › mdr/MDR020.pdf]

MDR021

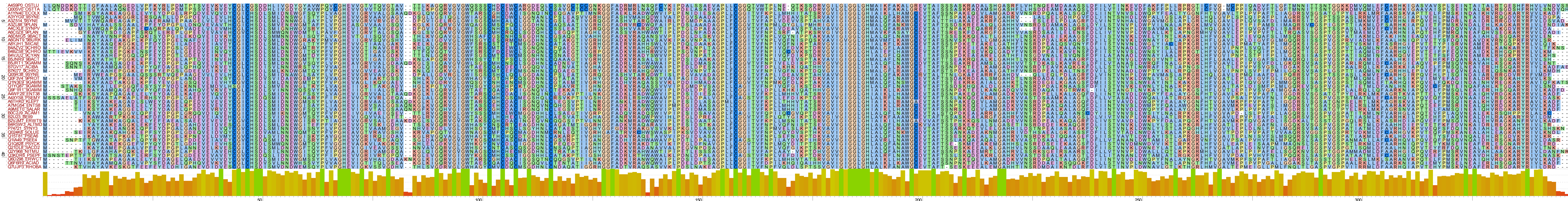

Supplement: Additional file 5 — Species distribution in MDR families. The numerical data underlying Figure 4 as a fixed width plain text text file of n(n/N) values where n denotes the number of seed sequences from the evolutionary group in question and N is the size of the corresponding seed set. [file 1471-2105-11-534-S5.ZIP › mdr/MDR021.pdf]

MDR023

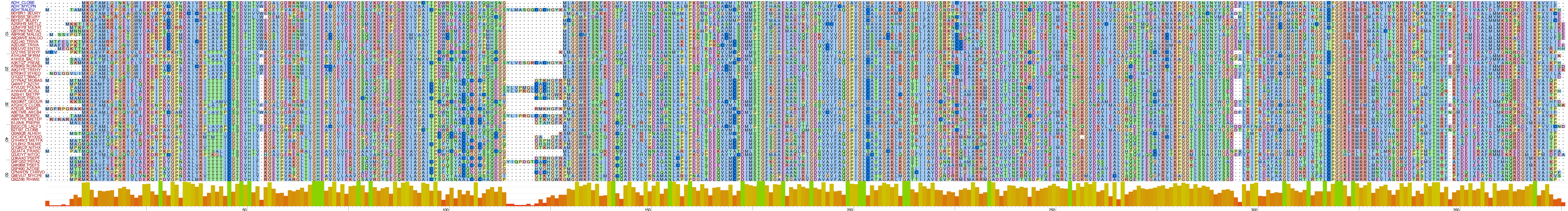

Supplement: Additional file 5 — Species distribution in MDR families. The numerical data underlying Figure 4 as a fixed width plain text text file of n(n/N) values where n denotes the number of seed sequences from the evolutionary group in question and N is the size of the corresponding seed set. [file 1471-2105-11-534-S5.ZIP › mdr/MDR023.pdf]

MDR024

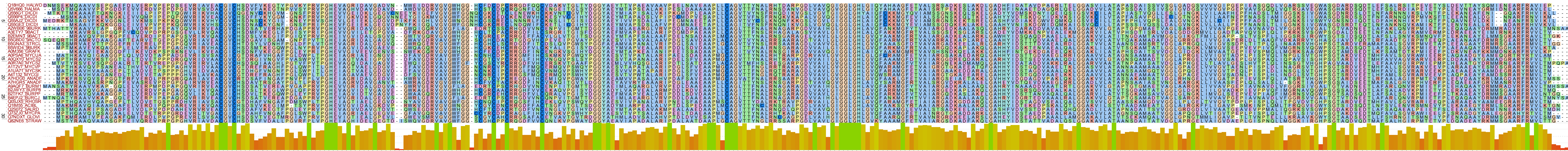

Supplement: Additional file 5 — Species distribution in MDR families. The numerical data underlying Figure 4 as a fixed width plain text text file of n(n/N) values where n denotes the number of seed sequences from the evolutionary group in question and N is the size of the corresponding seed set. [file 1471-2105-11-534-S5.ZIP › mdr/MDR024.pdf]

MDR025

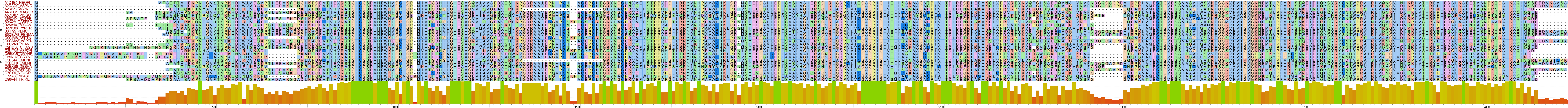

Supplement: Additional file 5 — Species distribution in MDR families. The numerical data underlying Figure 4 as a fixed width plain text text file of n(n/N) values where n denotes the number of seed sequences from the evolutionary group in question and N is the size of the corresponding seed set. [file 1471-2105-11-534-S5.ZIP › mdr/MDR025.pdf]

MDR027

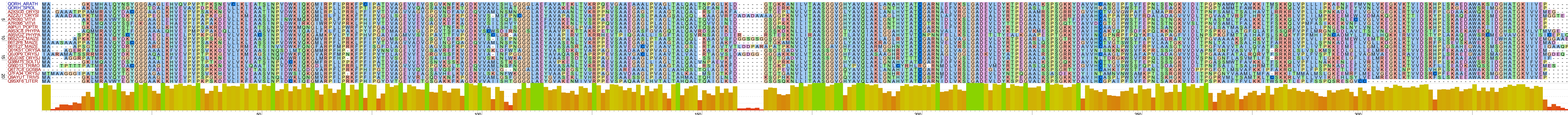

Supplement: Additional file 5 — Species distribution in MDR families. The numerical data underlying Figure 4 as a fixed width plain text text file of n(n/N) values where n denotes the number of seed sequences from the evolutionary group in question and N is the size of the corresponding seed set. [file 1471-2105-11-534-S5.ZIP › mdr/MDR027.pdf]

MDR030

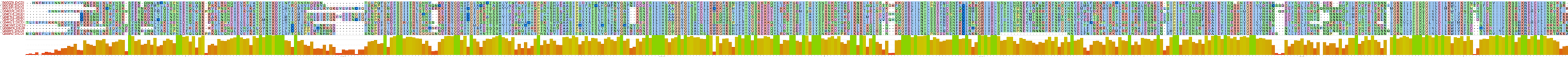

Supplement: Additional file 5 — Species distribution in MDR families. The numerical data underlying Figure 4 as a fixed width plain text text file of n(n/N) values where n denotes the number of seed sequences from the evolutionary group in question and N is the size of the corresponding seed set. [file 1471-2105-11-534-S5.ZIP › mdr/MDR030.pdf]

MDR031

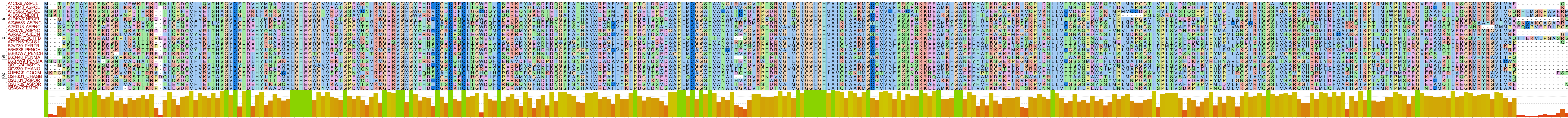

Supplement: Additional file 5 — Species distribution in MDR families. The numerical data underlying Figure 4 as a fixed width plain text text file of n(n/N) values where n denotes the number of seed sequences from the evolutionary group in question and N is the size of the corresponding seed set. [file 1471-2105-11-534-S5.ZIP › mdr/MDR031.pdf]

MDR033

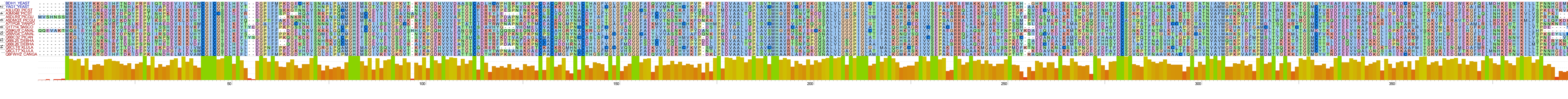

Supplement: Additional file 5 — Species distribution in MDR families. The numerical data underlying Figure 4 as a fixed width plain text text file of n(n/N) values where n denotes the number of seed sequences from the evolutionary group in question and N is the size of the corresponding seed set. [file 1471-2105-11-534-S5.ZIP › mdr/MDR033.pdf]

# MDR034

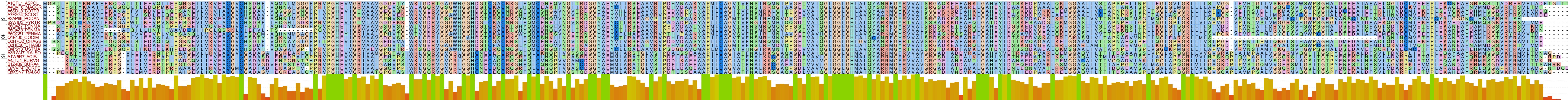

Supplement: Additional file 5 — Species distribution in MDR families. The numerical data underlying Figure 4 as a fixed width plain text text file of n(n/N) values where n denotes the number of seed sequences from the evolutionary group in question and N is the size of the corresponding seed set. [file 1471-2105-11-534-S5.ZIP › mdr/MDR034.pdf]

# MDR036

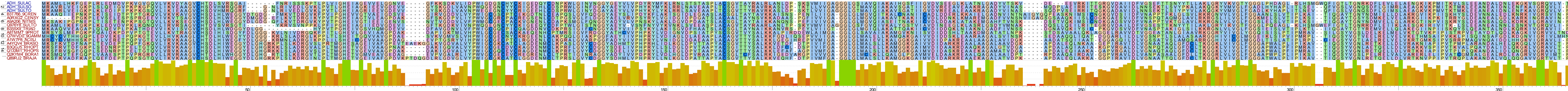

Supplement: Additional file 5 — Species distribution in MDR families. The numerical data underlying Figure 4 as a fixed width plain text text file of n(n/N) values where n denotes the number of seed sequences from the evolutionary group in question and N is the size of the corresponding seed set. [file 1471-2105-11-534-S5.ZIP › mdr/MDR036.pdf]

MDR037

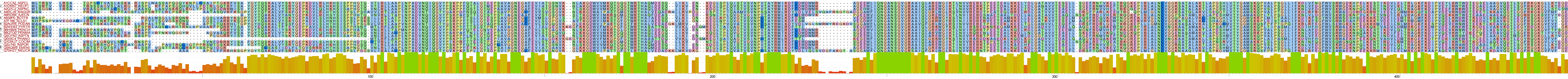

Supplement: Additional file 5 — Species distribution in MDR families. The numerical data underlying Figure 4 as a fixed width plain text text file of n(n/N) values where n denotes the number of seed sequences from the evolutionary group in question and N is the size of the corresponding seed set. [file 1471-2105-11-534-S5.ZIP › mdr/MDR037.pdf]

# MDR038

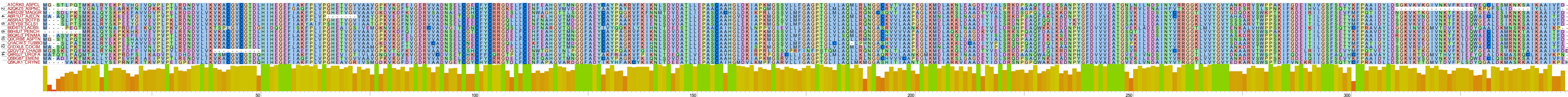

Supplement: Additional file 5 — Species distribution in MDR families. The numerical data underlying Figure 4 as a fixed width plain text text file of n(n/N) values where n denotes the number of seed sequences from the evolutionary group in question and N is the size of the corresponding seed set. [file 1471-2105-11-534-S5.ZIP › mdr/MDR038.pdf]

## MDR040

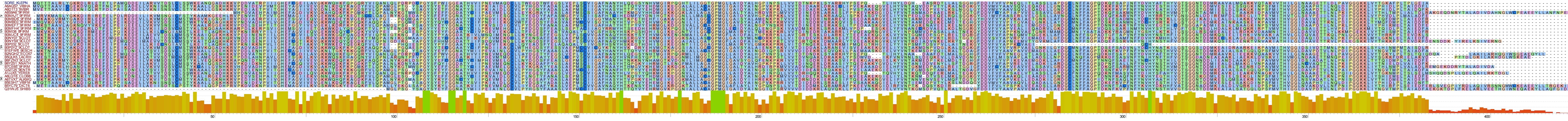

Supplement: Additional file 5 — Species distribution in MDR families. The numerical data underlying Figure 4 as a fixed width plain text text file of n(n/N) values where n denotes the number of seed sequences from the evolutionary group in question and N is the size of the corresponding seed set. [file 1471-2105-11-534-S5.ZIP › mdr/MDR040.pdf]

MDR041

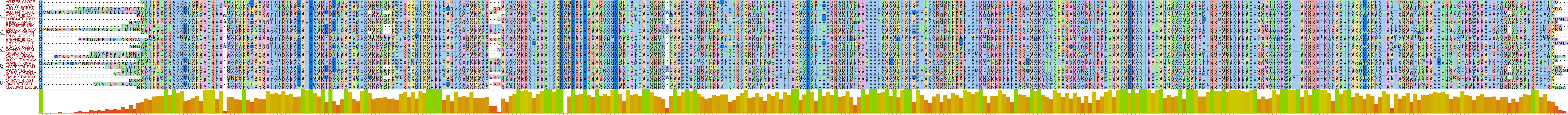

Supplement: Additional file 5 — Species distribution in MDR families. The numerical data underlying Figure 4 as a fixed width plain text text file of n(n/N) values where n denotes the number of seed sequences from the evolutionary group in question and N is the size of the corresponding seed set. [file 1471-2105-11-534-S5.ZIP › mdr/MDR041.pdf]

MDR042

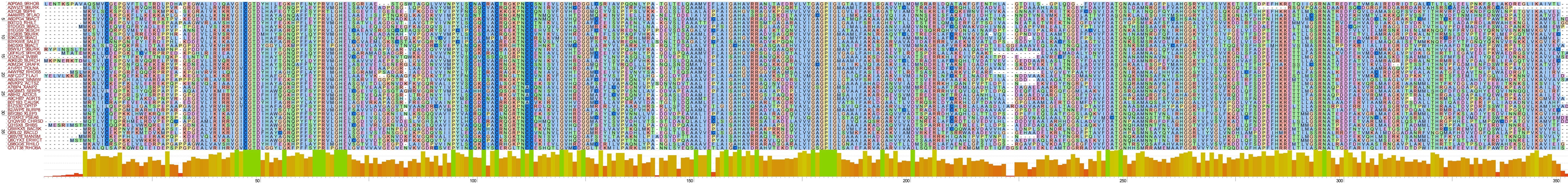

Supplement: Additional file 5 — Species distribution in MDR families. The numerical data underlying Figure 4 as a fixed width plain text text file of n(n/N) values where n denotes the number of seed sequences from the evolutionary group in question and N is the size of the corresponding seed set. [file 1471-2105-11-534-S5.ZIP › mdr/MDR042.pdf]

MDR044

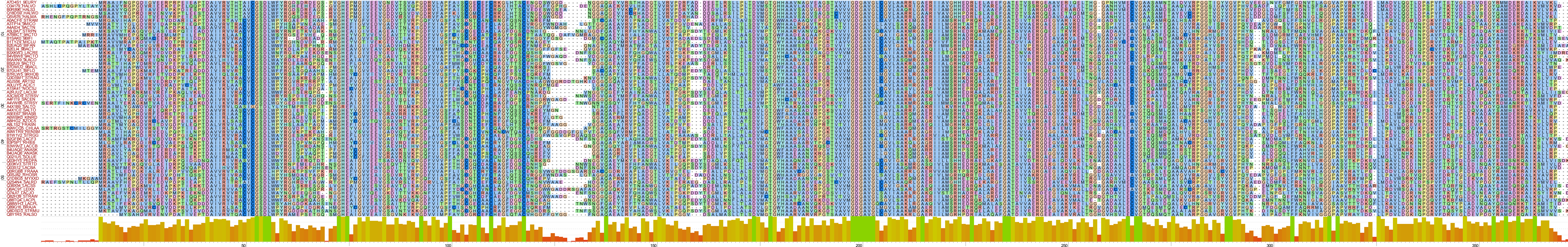

Supplement: Additional file 5 — Species distribution in MDR families. The numerical data underlying Figure 4 as a fixed width plain text text file of n(n/N) values where n denotes the number of seed sequences from the evolutionary group in question and N is the size of the corresponding seed set. [file 1471-2105-11-534-S5.ZIP › mdr/MDR044.pdf]

## MDR045

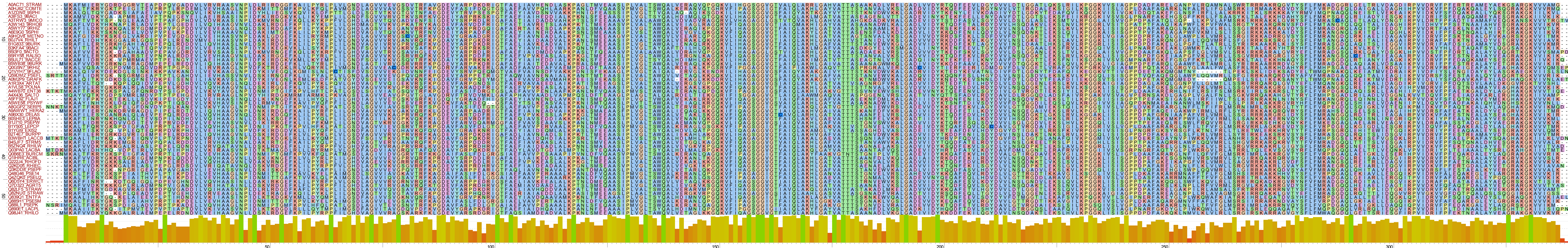

Supplement: Additional file 5 — Species distribution in MDR families. The numerical data underlying Figure 4 as a fixed width plain text text file of n(n/N) values where n denotes the number of seed sequences from the evolutionary group in question and N is the size of the corresponding seed set. [file 1471-2105-11-534-S5.ZIP › mdr/MDR045.pdf]

# MDR046

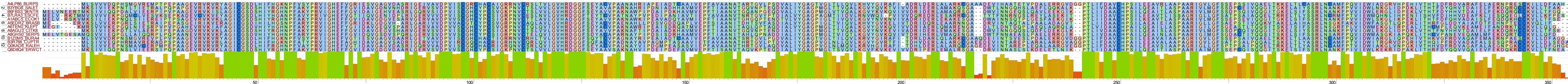

Supplement: Additional file 5 — Species distribution in MDR families. The numerical data underlying Figure 4 as a fixed width plain text text file of n(n/N) values where n denotes the number of seed sequences from the evolutionary group in question and N is the size of the corresponding seed set. [file 1471-2105-11-534-S5.ZIP › mdr/MDR046.pdf]

MDR047

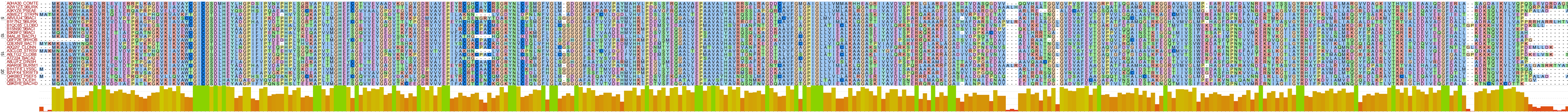

Supplement: Additional file 5 — Species distribution in MDR families. The numerical data underlying Figure 4 as a fixed width plain text text file of n(n/N) values where n denotes the number of seed sequences from the evolutionary group in question and N is the size of the corresponding seed set. [file 1471-2105-11-534-S5.ZIP › mdr/MDR047.pdf]

# MDR048

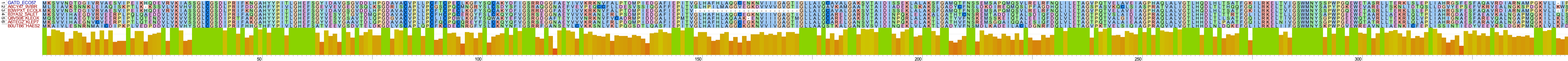

Supplement: Additional file 5 — Species distribution in MDR families. The numerical data underlying Figure 4 as a fixed width plain text text file of n(n/N) values where n denotes the number of seed sequences from the evolutionary group in question and N is the size of the corresponding seed set. [file 1471-2105-11-534-S5.ZIP › mdr/MDR048.pdf]

MDR049

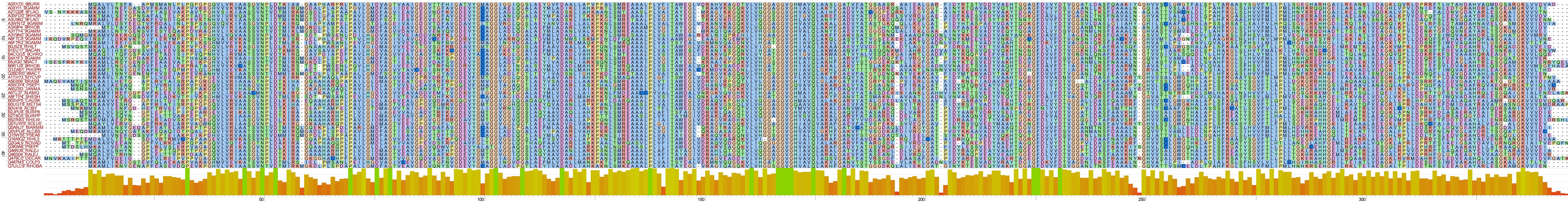

Supplement: Additional file 5 — Species distribution in MDR families. The numerical data underlying Figure 4 as a fixed width plain text text file of n(n/N) values where n denotes the number of seed sequences from the evolutionary group in question and N is the size of the corresponding seed set. [file 1471-2105-11-534-S5.ZIP › mdr/MDR049.pdf]

MDR050

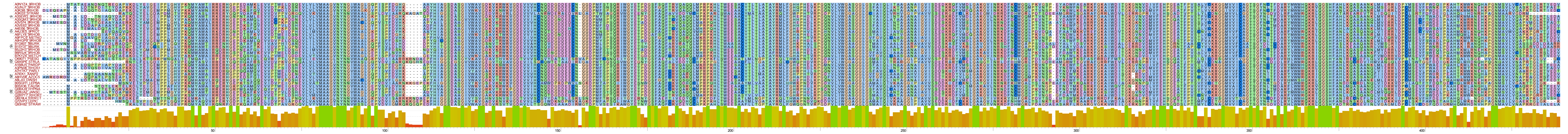

Supplement: Additional file 5 — Species distribution in MDR families. The numerical data underlying Figure 4 as a fixed width plain text text file of n(n/N) values where n denotes the number of seed sequences from the evolutionary group in question and N is the size of the corresponding seed set. [file 1471-2105-11-534-S5.ZIP › mdr/MDR050.pdf]

# MDR051

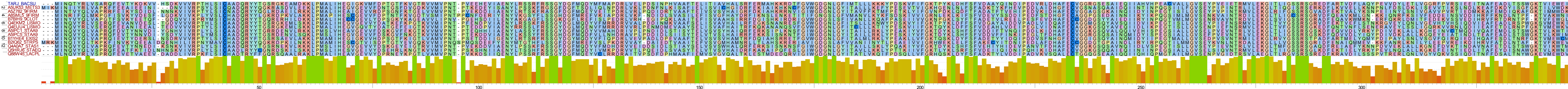

Supplement: Additional file 5 — Species distribution in MDR families. The numerical data underlying Figure 4 as a fixed width plain text text file of n(n/N) values where n denotes the number of seed sequences from the evolutionary group in question and N is the size of the corresponding seed set. [file 1471-2105-11-534-S5.ZIP › mdr/MDR051.pdf]

# MDR052

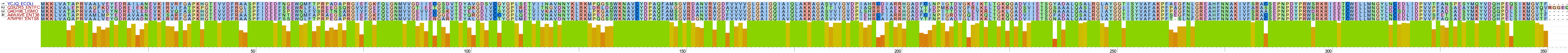

Supplement: Additional file 5 — Species distribution in MDR families. The numerical data underlying Figure 4 as a fixed width plain text text file of n(n/N) values where n denotes the number of seed sequences from the evolutionary group in question and N is the size of the corresponding seed set. [file 1471-2105-11-534-S5.ZIP › mdr/MDR052.pdf]

# MDR054

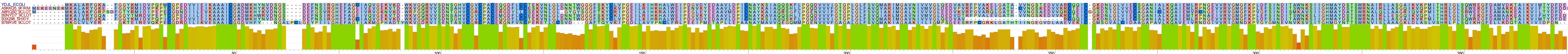

Supplement: Additional file 5 — Species distribution in MDR families. The numerical data underlying Figure 4 as a fixed width plain text text file of n(n/N) values where n denotes the number of seed sequences from the evolutionary group in question and N is the size of the corresponding seed set. [file 1471-2105-11-534-S5.ZIP › mdr/MDR054.pdf]

MDR055

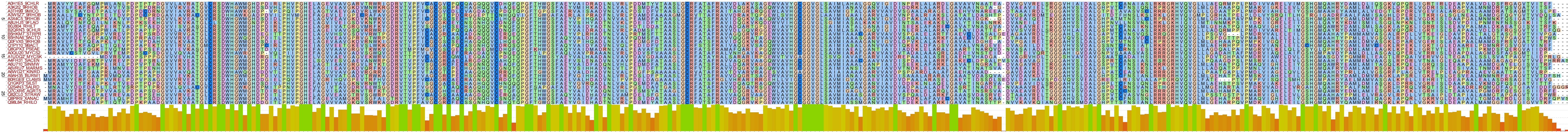

Supplement: Additional file 5 — Species distribution in MDR families. The numerical data underlying Figure 4 as a fixed width plain text text file of n(n/N) values where n denotes the number of seed sequences from the evolutionary group in question and N is the size of the corresponding seed set. [file 1471-2105-11-534-S5.ZIP › mdr/MDR055.pdf]

# MDR056

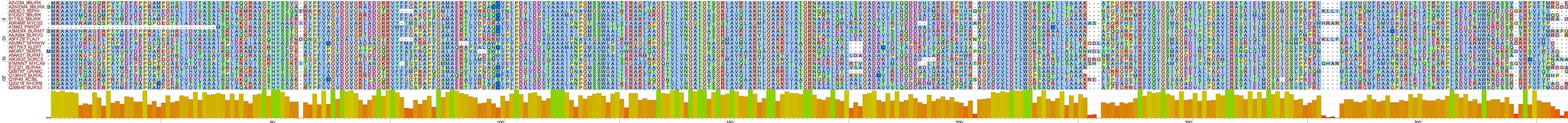

Supplement: Additional file 5 — Species distribution in MDR families. The numerical data underlying Figure 4 as a fixed width plain text text file of n(n/N) values where n denotes the number of seed sequences from the evolutionary group in question and N is the size of the corresponding seed set. [file 1471-2105-11-534-S5.ZIP › mdr/MDR056.pdf]

## MDR057

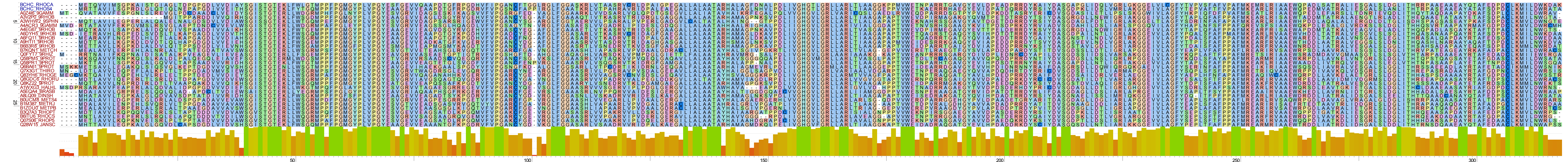

Supplement: Additional file 5 — Species distribution in MDR families. The numerical data underlying Figure 4 as a fixed width plain text text file of n(n/N) values where n denotes the number of seed sequences from the evolutionary group in question and N is the size of the corresponding seed set. [file 1471-2105-11-534-S5.ZIP › mdr/MDR057.pdf]

# MDR058

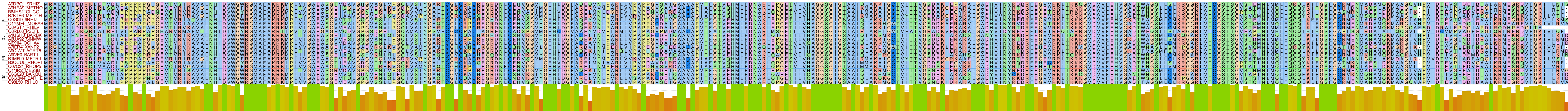

Supplement: Additional file 5 — Species distribution in MDR families. The numerical data underlying Figure 4 as a fixed width plain text text file of n(n/N) values where n denotes the number of seed sequences from the evolutionary group in question and N is the size of the corresponding seed set. [file 1471-2105-11-534-S5.ZIP › mdr/MDR058.pdf]

MDR059

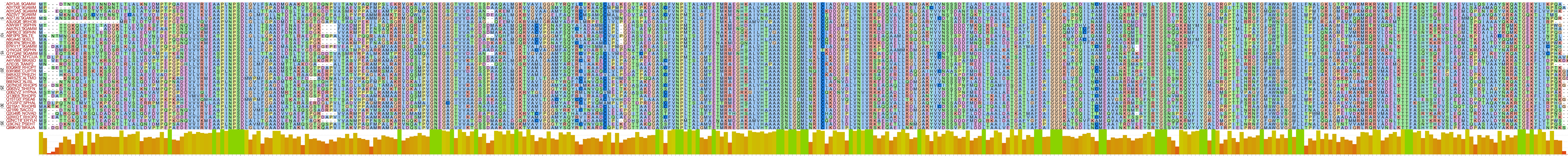

Supplement: Additional file 5 — Species distribution in MDR families. The numerical data underlying Figure 4 as a fixed width plain text text file of n(n/N) values where n denotes the number of seed sequences from the evolutionary group in question and N is the size of the corresponding seed set. [file 1471-2105-11-534-S5.ZIP › mdr/MDR059.pdf]

# MDR060

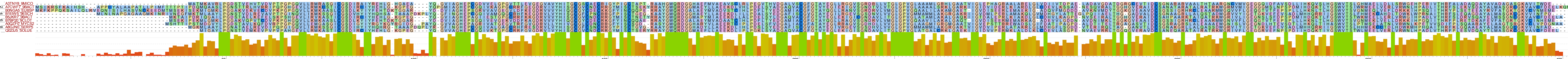

Supplement: Additional file 5 — Species distribution in MDR families. The numerical data underlying Figure 4 as a fixed width plain text text file of n(n/N) values where n denotes the number of seed sequences from the evolutionary group in question and N is the size of the corresponding seed set. [file 1471-2105-11-534-S5.ZIP › mdr/MDR060.pdf]

# MDR061

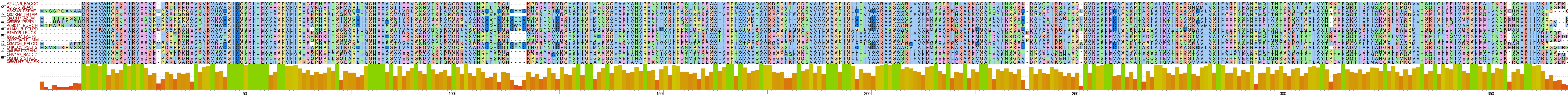

Supplement: Additional file 5 — Species distribution in MDR families. The numerical data underlying Figure 4 as a fixed width plain text text file of n(n/N) values where n denotes the number of seed sequences from the evolutionary group in question and N is the size of the corresponding seed set. [file 1471-2105-11-534-S5.ZIP › mdr/MDR061.pdf]

MDR062

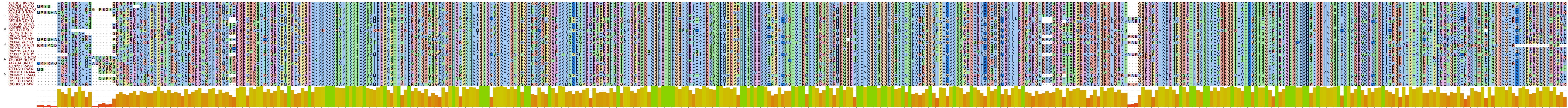

Supplement: Additional file 5 — Species distribution in MDR families. The numerical data underlying Figure 4 as a fixed width plain text text file of n(n/N) values where n denotes the number of seed sequences from the evolutionary group in question and N is the size of the corresponding seed set. [file 1471-2105-11-534-S5.ZIP › mdr/MDR062.pdf]

# MDR063

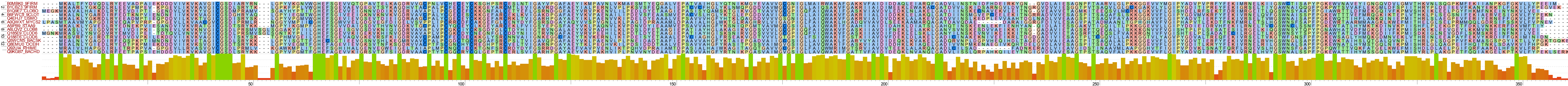

Supplement: Additional file 5 — Species distribution in MDR families. The numerical data underlying Figure 4 as a fixed width plain text text file of n(n/N) values where n denotes the number of seed sequences from the evolutionary group in question and N is the size of the corresponding seed set. [file 1471-2105-11-534-S5.ZIP › mdr/MDR063.pdf]

MDR064

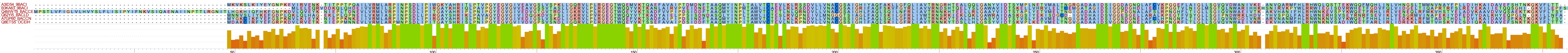

Supplement: Additional file 5 — Species distribution in MDR families. The numerical data underlying Figure 4 as a fixed width plain text text file of n(n/N) values where n denotes the number of seed sequences from the evolutionary group in question and N is the size of the corresponding seed set. [file 1471-2105-11-534-S5.ZIP › mdr/MDR064.pdf]

## MDR065

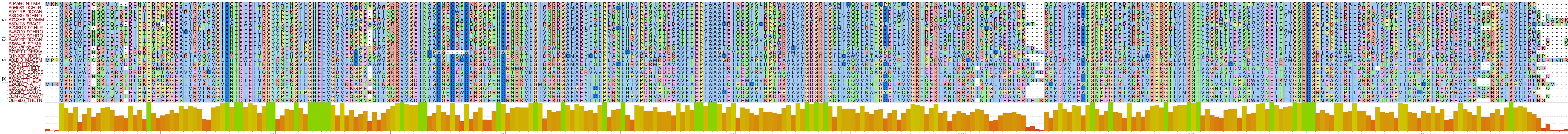

Supplement: Additional file 5 — Species distribution in MDR families. The numerical data underlying Figure 4 as a fixed width plain text text file of n(n/N) values where n denotes the number of seed sequences from the evolutionary group in question and N is the size of the corresponding seed set. [file 1471-2105-11-534-S5.ZIP › mdr/MDR065.pdf]

## MDR066

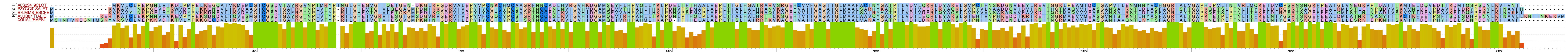

Supplement: Additional file 5 — Species distribution in MDR families. The numerical data underlying Figure 4 as a fixed width plain text text file of n(n/N) values where n denotes the number of seed sequences from the evolutionary group in question and N is the size of the corresponding seed set. [file 1471-2105-11-534-S5.ZIP › mdr/MDR066.pdf]

## MDR067

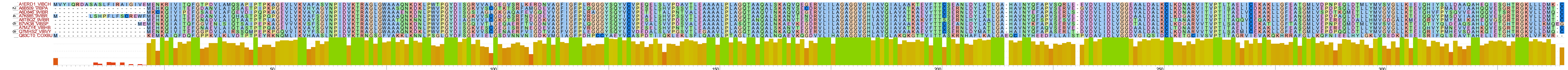

Supplement: Additional file 5 — Species distribution in MDR families. The numerical data underlying Figure 4 as a fixed width plain text text file of n(n/N) values where n denotes the number of seed sequences from the evolutionary group in question and N is the size of the corresponding seed set. [file 1471-2105-11-534-S5.ZIP › mdr/MDR067.pdf]

MDR068

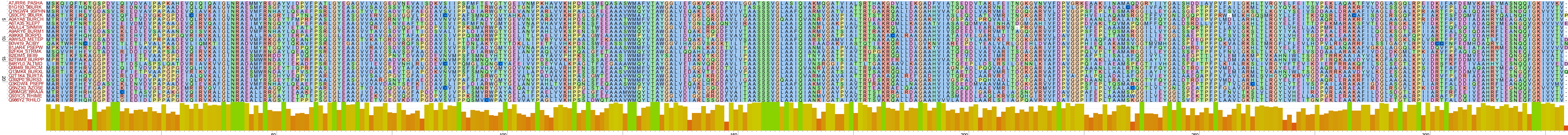

Supplement: Additional file 5 — Species distribution in MDR families. The numerical data underlying Figure 4 as a fixed width plain text text file of n(n/N) values where n denotes the number of seed sequences from the evolutionary group in question and N is the size of the corresponding seed set. [file 1471-2105-11-534-S5.ZIP › mdr/MDR068.pdf]

# MDR069

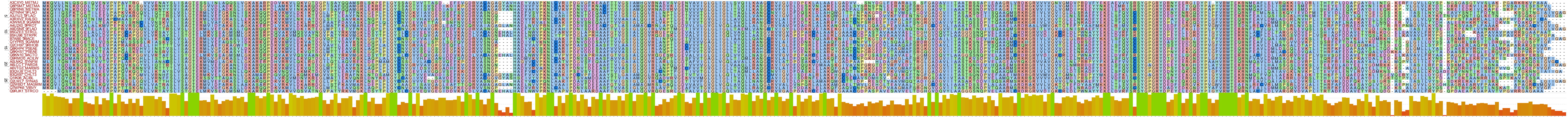

Supplement: Additional file 5 — Species distribution in MDR families. The numerical data underlying Figure 4 as a fixed width plain text text file of n(n/N) values where n denotes the number of seed sequences from the evolutionary group in question and N is the size of the corresponding seed set. [file 1471-2105-11-534-S5.ZIP › mdr/MDR069.pdf]

MDR070

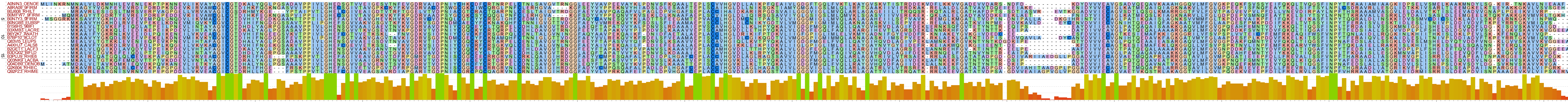

Supplement: Additional file 5 — Species distribution in MDR families. The numerical data underlying Figure 4 as a fixed width plain text text file of n(n/N) values where n denotes the number of seed sequences from the evolutionary group in question and N is the size of the corresponding seed set. [file 1471-2105-11-534-S5.ZIP › mdr/MDR070.pdf]

MDR071

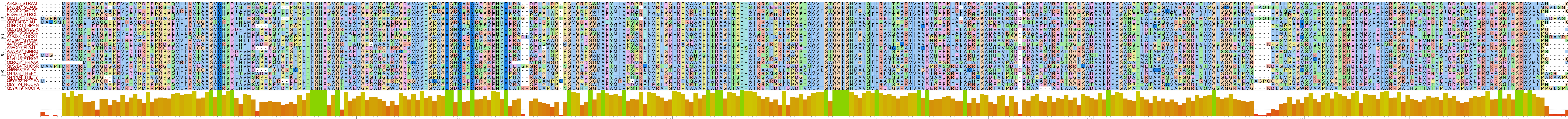

Supplement: Additional file 5 — Species distribution in MDR families. The numerical data underlying Figure 4 as a fixed width plain text text file of n(n/N) values where n denotes the number of seed sequences from the evolutionary group in question and N is the size of the corresponding seed set. [file 1471-2105-11-534-S5.ZIP › mdr/MDR071.pdf]

# MDR072

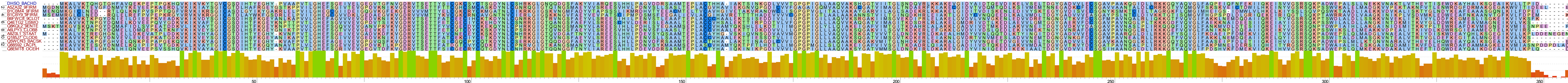

Supplement: Additional file 5 — Species distribution in MDR families. The numerical data underlying Figure 4 as a fixed width plain text text file of n(n/N) values where n denotes the number of seed sequences from the evolutionary group in question and N is the size of the corresponding seed set. [file 1471-2105-11-534-S5.ZIP › mdr/MDR072.pdf]

# MDR073

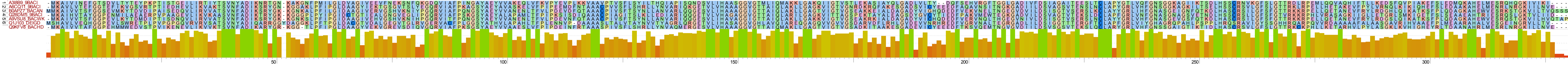

Supplement: Additional file 5 — Species distribution in MDR families. The numerical data underlying Figure 4 as a fixed width plain text text file of n(n/N) values where n denotes the number of seed sequences from the evolutionary group in question and N is the size of the corresponding seed set. [file 1471-2105-11-534-S5.ZIP › mdr/MDR073.pdf]

# MDR074

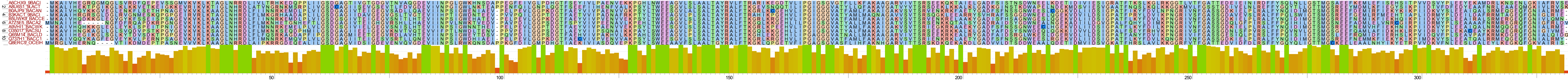

Supplement: Additional file 5 — Species distribution in MDR families. The numerical data underlying Figure 4 as a fixed width plain text text file of n(n/N) values where n denotes the number of seed sequences from the evolutionary group in question and N is the size of the corresponding seed set. [file 1471-2105-11-534-S5.ZIP › mdr/MDR074.pdf]

MDR075

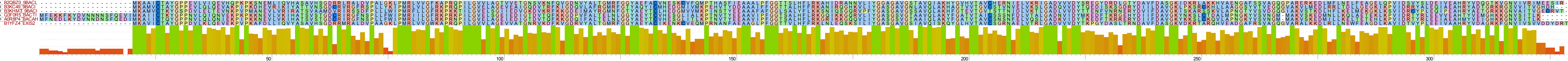

Supplement: Additional file 5 — Species distribution in MDR families. The numerical data underlying Figure 4 as a fixed width plain text text file of n(n/N) values where n denotes the number of seed sequences from the evolutionary group in question and N is the size of the corresponding seed set. [file 1471-2105-11-534-S5.ZIP › mdr/MDR075.pdf]

MDR076

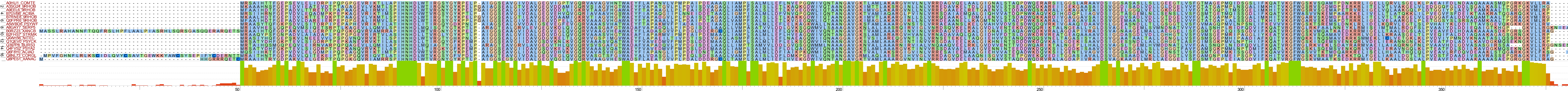

Supplement: Additional file 5 — Species distribution in MDR families. The numerical data underlying Figure 4 as a fixed width plain text text file of n(n/N) values where n denotes the number of seed sequences from the evolutionary group in question and N is the size of the corresponding seed set. [file 1471-2105-11-534-S5.ZIP › mdr/MDR076.pdf]

MDR078

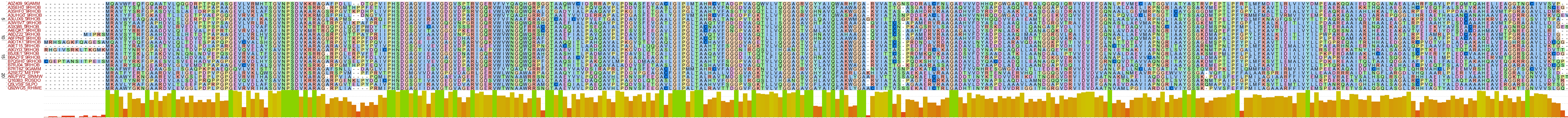

Supplement: Additional file 5 — Species distribution in MDR families. The numerical data underlying Figure 4 as a fixed width plain text text file of n(n/N) values where n denotes the number of seed sequences from the evolutionary group in question and N is the size of the corresponding seed set. [file 1471-2105-11-534-S5.ZIP › mdr/MDR078.pdf]

# MDR080

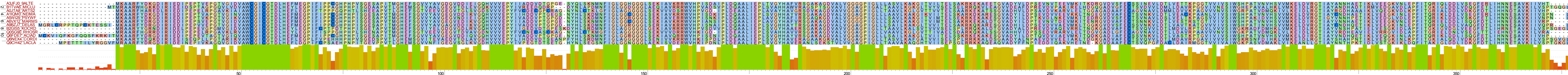

Supplement: Additional file 5 — Species distribution in MDR families. The numerical data underlying Figure 4 as a fixed width plain text text file of n(n/N) values where n denotes the number of seed sequences from the evolutionary group in question and N is the size of the corresponding seed set. [file 1471-2105-11-534-S5.ZIP › mdr/MDR080.pdf]

# MDR081

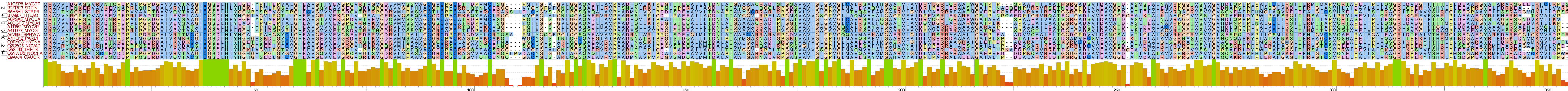

Supplement: Additional file 5 — Species distribution in MDR families. The numerical data underlying Figure 4 as a fixed width plain text text file of n(n/N) values where n denotes the number of seed sequences from the evolutionary group in question and N is the size of the corresponding seed set. [file 1471-2105-11-534-S5.ZIP › mdr/MDR081.pdf]

# MDR083

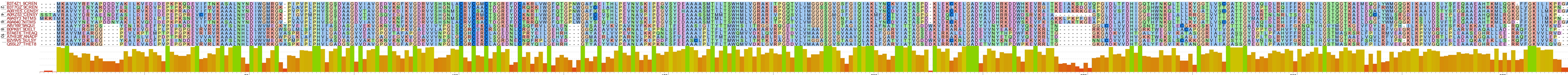

Supplement: Additional file 5 — Species distribution in MDR families. The numerical data underlying Figure 4 as a fixed width plain text text file of n(n/N) values where n denotes the number of seed sequences from the evolutionary group in question and N is the size of the corresponding seed set. [file 1471-2105-11-534-S5.ZIP › mdr/MDR083.pdf]

# MDR085

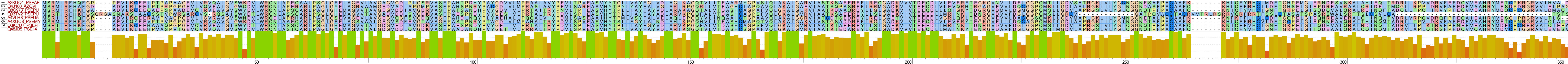

Supplement: Additional file 5 — Species distribution in MDR families. The numerical data underlying Figure 4 as a fixed width plain text text file of n(n/N) values where n denotes the number of seed sequences from the evolutionary group in question and N is the size of the corresponding seed set. [file 1471-2105-11-534-S5.ZIP › mdr/MDR085.pdf]

MDR086

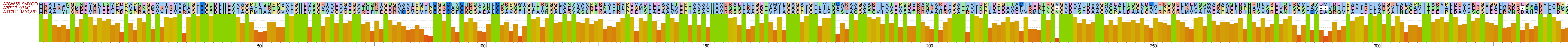

Supplement: Additional file 5 — Species distribution in MDR families. The numerical data underlying Figure 4 as a fixed width plain text text file of n(n/N) values where n denotes the number of seed sequences from the evolutionary group in question and N is the size of the corresponding seed set. [file 1471-2105-11-534-S5.ZIP › mdr/MDR086.pdf]
